# Synthesis, Anti-Cancer Activity, Cell Cycle Arrest, Apoptosis Induction, and Docking Study of Fused Benzo[*h*]chromeno[2,3-*d*]pyrimidine on Human Breast Cancer Cell Line MCF-7

**DOI:** 10.3390/molecules29194697

**Published:** 2024-10-04

**Authors:** Zainab M. Khoder, Mosaad S. Mohamed, Samir M. Awad, Amal F. Gharib, Omnia Aly, Marwa Abd El-Fattah Khodair, Samar S. Fatahala, Rania H. Abd El-Hameed

**Affiliations:** 1 Pharmaceutical Organic Chemistry Department, Faculty of Pharmacy, Helwan University, Helwan, Cairo 11795, Egypt; zkhoder@buffalo.edu (Z.M.K.); mosaad_abdlallah@pharm.helwan.edu.eg (M.S.M.); samirawad2000@yahoo.com (S.M.A.); zeiadomar@yahoo.com (R.H.A.E.-H.); 2 Department of Chemistry, The State University of New York at Buffalo, New York, NY 14260, USA; 3 Pharmacy Department, Al-zahrawi University College, Carbala 56001, Iraq; 4 Department of Clinical Laboratory Sciences, College of Applied Medical Sciences, Taif University, Taif 21944, Saudi Arabia; dr.amal.f.gharib@gmail.com; 5 Medical Biochemistry Department, National Research Centre, Doki, P.O. Box 12622 Cairo, Egypt; oe.el-sayed@nrc.sci.eg

**Keywords:** chromenopyrimidine, NCI-assay, cytotoxicity, molecular docking, Bcl-2/Mcl-1 assay, apoptosis

## Abstract

Breast cancer is the predominant form of cancer among women and ranks as the second most prevalent cancer globally, affecting both developed and less developed countries. Presently, accessible cancer treatment methods either employ recently created, secure, and efficient chemotherapeutic medications or directly target innovative pathways that cause apoptosis. One of the indirect strategies for treating this fatal illness has mostly depended on its essential role in cell cycle arrest and apoptosis induction, as well as the antagonistic interaction between the Bcl-2 and Mcl-1 proteins, in order to avert major health repercussions. We reported that newly synthesized fused chromenopyrimidines (**3a** and **4a**) showed potential cell cycle arrest and dual Bcl-2 and Mcl-1 inhibitory characteristics. Bcl-2 and Mcl-1 were the targets of a molecular docking procedure. The previous docking results are in line with the biological data and suggest that **3a** may have promising anti-cancer activity.

## 1. Introduction

Uncontrolled growth “cancer” is the world’s second leading cause of death, following cardiovascular disorders [1,2]. Breast cancer is a fatal disease caused by aberrant breast cells that proliferate and develop into tumors that become deadly if they spread throughout the body (metastasize) and are not treated. According to the WHO, 2.3 million women worldwide had a breast cancer diagnosis in 2022, and 670,000 people died from this disease [3]. Resistance was found in the chemotherapy drugs that were previously used to treat metastatic breast cancer [4]. Discovering anti-cancer medications with reduced resistance and increased selectivity and potency, particularly for breast cancer, is the scientists’ imminent task. They turned to creating medications by synthesizing substances that accelerate apoptosis and/or inhibit the cell cycle and employing them either in place of or in addition to conventional medications [5,6,7,8].

Apoptosis is a programmed cell death that occurs through either the extrinsic pathway [cell surface death receptors] or intrinsic pathway [invasion of mitochondria] [9,10]. Both intrinsic and extrinsic pathways lead to DNA damage and allow the cells to pass away with intact membranes, which limits inflammatory responses [11].

The main regulator of intrinsic pathway is the B cell lymphoma 2 (Bcl-2) family. The Bcl-2 family, according to their morphology and role in apoptosis, are subdivided into three groups: the antiapoptotic proteins (Bcl-2, Bcl-xL, BclW, and myeloid cell leukemia-1 (Mcl-1)), the sensor’s proapoptotic BH3—only proteins (BIM, BMF, and PUMA), and the executor’s proapoptotic (BAX and BAK). As an antiapoptotic member of the Bcl-2 family, Mcl-1 is characterized as a rapid sensor that regulates cell death and cell cycle progression and mitochondrial homeostasis, as well as contributing to cell resistance to diverse chemotherapeutic agents [12,13].

**Figure 1:**
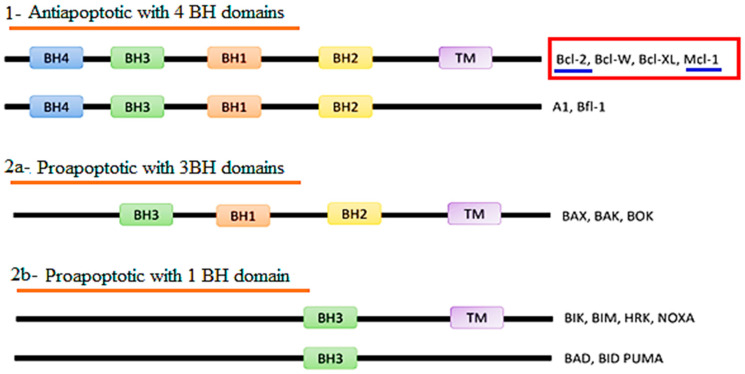
Classification of the Bcl-2 protein family according to Bcl-2 homology (BH) domains.

The Bcl-2 family are characterized by the presence of Bcl-2 homology (BH) domains, as well as a carboxy-terminal transmembrane domain (TMD). Moreover, the number of BH domains also aids in categorization of Bcl-2 groups, with Bcl-2 and Mcl-1 both sharing three BH regions [14,15,16] (Figure 1).

**Figure 2:**
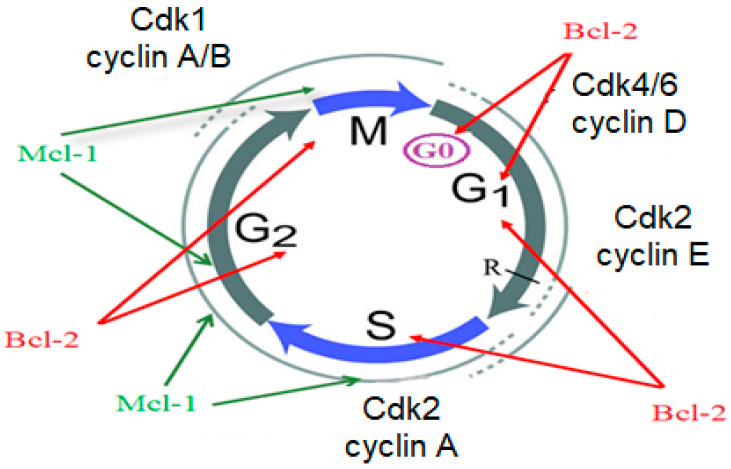
Role of Bcl-2 and Mcl-1 in cell cycle induction.

Furthermore, the role of Bcl-2 and Mcl-1 is not limited to apoptosis; they play a vital role in cell cycle progression, especially in breast cancer. In detail, Bcl-2 affects the cell cycle in its G0/G1 check point, G2/M, and G1/S phases, while Mcl-1 interacts with cell cycle kinases, specifically CDK2 as well as CDK4, and, hence, disturbs the cycle at their G2/M and S/G2 phases [17,18,19,20] (Figure 2).

Over the past few years, numerous Bcl-2 protein family inhibitors have been produced, such as obatoclax (GX15-070), venetoclax (ABT-199), navitoclax (ABT-263), and oblimersen sodium (G3139) [16,21,22]. Also, there are compounds in clinical trials that have the best binding to Mcl-1 like A-1210477 [23,24], AZD5991 [25], and ABBV 462 [26]. According to the ligand/protein complex analysis, the most significant Mcl-1 hot-spots are the P2 and P3 pockets, as well as the crucial polar interaction with Arg 263, whereas the P2 and P4 pockets are the most significant Bcl-2 hot-spots [16,27,28,29,30]. Furthermore, as concluded from recent study that indicated that hundreds of homomorphic antagonists mimic the Bcl-2 BH3 domain, they must interact with the hydrophobic structure of Bcl-2, where any modification on the hydrophobic structure leads to the inactivation and the loss of ability of anti-apoptotic Bcl-2, to bind to other members to form dimers [20].

**Figure 3:**
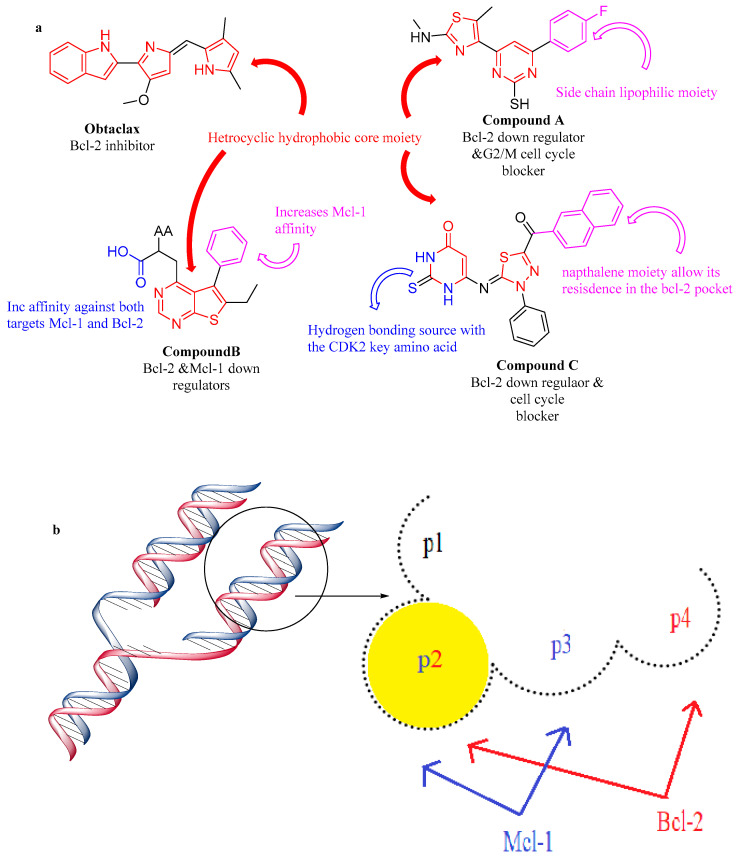
Structure features of Bcl-2 and Mcl-1 protein/antagonist. (**a**) Structure activity relationship of Bcl-2 and Mcl-1 inhibitors, (**b**) Structure features of Bcl-2 and Mcl-1 protein.

Therefore, the key of finding more selective and effective anti-cancer agents is occupying the P2 pocket with a small lipophilic compound (Figure 3).

**Figure 4:**
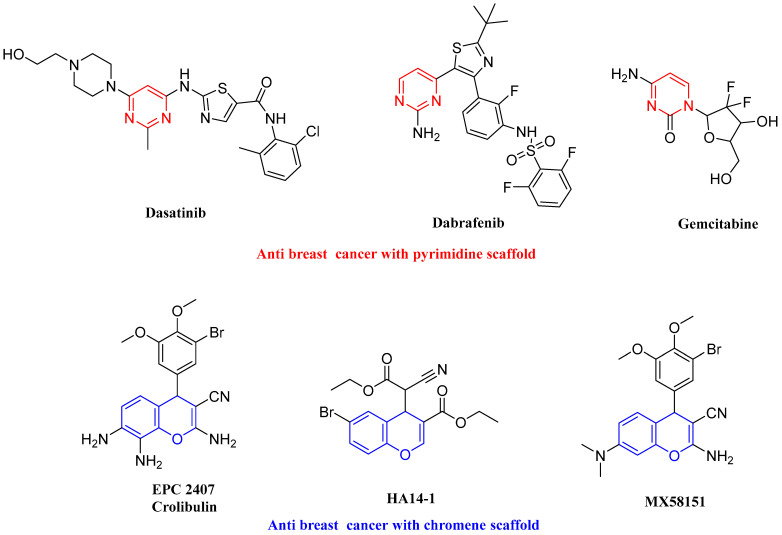
Drugs containing pyrimidine or chromenes.

Based on the notable effect of pyrimidines and chromenes in treating breast cancer [31,32,33,34,35,36,37,38,39,40] (Figure 4), their vital role in cell cycle arrest and apoptosis induction [41,42,43,44,45,46,47,48] and the previous finding about the structure features of Bcl-2 and Mcl-1 proteins/antagonist interaction, as well as the need for selecting less resistant drugs, our aim is to synthesize novel fused chromenopyrimidine with promising cell cycle arrest as well as dual Bcl-2 and Mcl-1 inhibitory activity through making some modifications in Bcl-2 and Mcl-1 candidates.

## 2. Results and Discussion

### 2.1. Chemistry

Our study’s objective was to synthesize various benzo[*h*]chromeno[2,3-*d*]pyrimidine derivatives and modify their structures in order to investigate the compounds’ antineoplastic properties and the impact of these structural changes on their biological activities. Therefore, we previously prepared 2-amino-4-aryl-4*H*-benzo[*h*]chromene-3-carbonitriles (**1a**–**c**) and 5-aryl-5*H*-benzo[*h*]chromeno[2,3-*d*]pyrimidine-4-ones (**2a**–**e**).

**Scheme 1:**
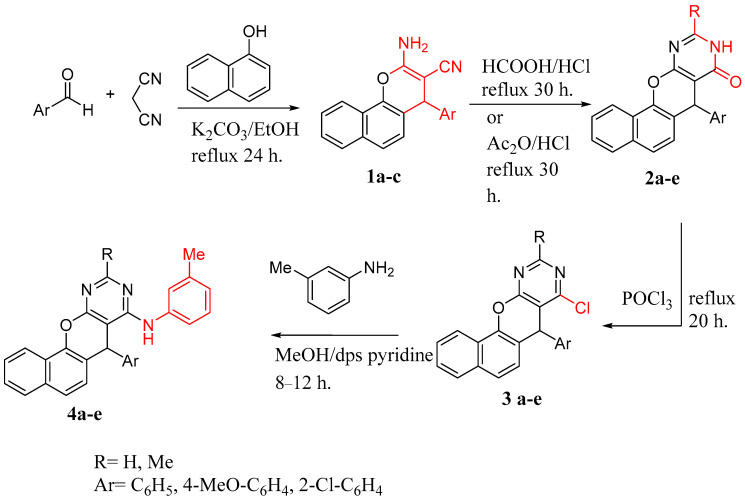
Synthetic pathway of compounds **1a**–**c** to **4 a**–**e.**

Due to the potency of benzo[*h*]chromene, as well as the use of a pyrimidine scaffold as an anti-cancer agent [47,49,50,51,52,53,54,55,56], we performed some structural modifications in compounds **2a**–**e** to obtain chlorobenzo[*h*]chromeno[2,3-*d*]pyrimidines and assessed their antitumor activity. According to the reported procedure [57], compounds **2a**–**e** were heated under reflux with phosphorous oxychloride to obtain 4-chloro derivative **3a**–**e**. Furthermore, to obtain 2-alkyl-5-aryl-*N*-(m-tolyl)-5*H*-benzo[*h*]chromeno[2,3-*d*]pyrimidine **4a**–**e**, compounds **3a**–**e** were separately heated under reflux with *meta*-toluidine in ethanol [58] (as revealed in Scheme 1). Spectral analysis verified all newly synthesized compounds.

### 2.2. Anti-Cancer Result and Discussion

#### 2.2.1. Single-Dose Testing

**Figure 5:**
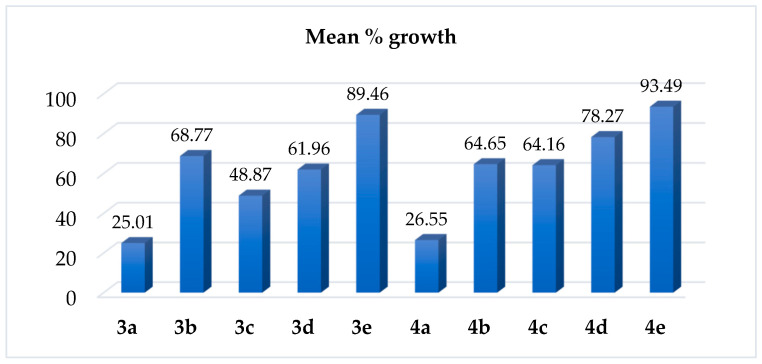
Mean % growth of **3a**–**e** and **4a**–**e**.

The newly synthesized compounds were selected by the National Cancer Institute for single-dose assay to determine their antineoplastic effect. The NCI single-dose testing results are presented in Table S1 and Figure 5.

The results reveal that two of these compounds (**3a** and **4a**), both bearing (***para*-methoxybenzyl moiety**), showed best cytotoxicity, making them qualified for further five-dose testing. In details, compound **3a (4-Chloro-5-(4-methoxyphenyl)-*5H*-benzo[*h*]chromeno[2,3-*d*]pyrimidine)** was lethal to nine cancer cell lines with % growth ranging from −51.24 to −2.39. Furthermore, a significant antitumor activity against 29 cell lines was exhibited by the same compound with % growth ranging from 1.98 to 32.43. It also recorded moderate activity toward 14 cell lines with % growth varying from 32.47 to 48.02. There was weak activity against seven cell lines with % growth ranging from 52.21 to 70.89. Compound **4a (5-(4-methoxyphenyl)-*N*-(m-tolyl)-*5H*-benzo[*h*]chromeno[2,3-*d*]pyrimidin-4-amine)** recoded complete cell death against seven cell lines with a range of −34.51 to −2.33%. Additionally, a remarkable effectiveness was demonstrated by the same molecule against 28 cell lines with a range of 1.00 to 30.88%; it also displayed a moderate effectiveness towards 14 cell lines with % growth varying from 33.05 to 48.44.

Compound **3b (4-Chloro-5-(2-chlorophenyl)-*5H*-benzo[*h*]chromeno[2,3-*d*]pyrimidine)** was found to exhibit noteworthy efficacy against four cell lines, with % growth ranging from 4.44 to 32.33. Additionally, this drug demonstrated moderate efficacy against eight cell lines with a range of 34.37 to 49.81 and total cell death against the melanoma cell line MDA-MB-435 with % growth of −37.42.

Compound **3c** (**4-Chloro-2-methyl-5-phenyl-*5H*-benzo[*h*]chromeno[2,3-*d*]pyrimidine)** showed significant activity against 16 cell lines with % growth ranging from 2.27 to 32.34, and it completely killed 2 cell lines, namely, NCI-H522 and MDA-MB-435 with % growth of −7.35 and −22.20, respectively. There, with a range of 32.70 to 49.93%, it demonstrated moderate activity against 15 cell lines.

Compound **3d (4-Chloro-5-(4-methoxyphenyl)-2-methyl-*5H*-benzo[*h*]chromeno[2,3-*d*]pyrimidine)** demonstrated substantial activity against six cell lines with growth % ranging from 13.50 to 27.74 and total cell death against two cell lines, specifically MDA-MB-435 and MDA-MB-468, with a % growth of −13.44 and −2.12, respectively. Also, it showed a moderate activity towards 13 cell lines with % growth ranging from 34.80 to 49.33. Finally, compound **3e (4-Chloro-5-(2-chlorophenyl)-2-methyl-*5H*-benzo[*h*]chromeno[2,3-*d*]pyrimidine)** exhibited moderate efficacy against renal cancer cell line CAKI-1, with a % growth of 50.18.

In the case of ***N*-tolyl amino derivative 4b**–**e**, compound **4b (5-(2-chlorophenyl)-*N*-(m-tolyl)-*5H*-benzo[*h*]chromeno[2,3-*d*]pyrimidin-4-amine)** demonstrated notable efficacy towards two cell lines, namely, K-562 and NCI-H522, demonstrating a % growth of 22.77 and 18.47, respectively. Additionally, it killed two cell lines, specifically MDA-MB-435 and MDA-MB-468 with a % growth of −40.67 and −13.09, respectively, and it showed moderate activity toward nine cell lines with % growth ranging from 34.05 to 48.41. Compound **4c** (**2-methyl-5-phenyl-*N*-(m-tolyl)-*5H*-benzo[*h*]chromeno[2,3-*d*]pyrimidin-4-amine)** demonstrated significant efficacy against six cell lines, with % growth ranging from 12.52 to 29.26. It also caused total cell death to the MDA-MB-435 melanoma cell line. Furthermore, the same molecule demonstrated moderate activity against eight cell lines with a range of 32.46 to 47.38%. Compound **4d (5-(4-methoxyphenyl)-2-methyl-*N*-(m-tolyl)-*5H*-benzo[*h*]chromeno[2,3-*d*]pyrimidin-4-amine)** demonstrated noteworthy activity against the melanoma MDA-MB-435 cell line and demonstrated moderate efficacy against K-562, SNB-75, and MCF-7 with a % growth of 39.52, 47.86, and 42.79, respectively, while compound **4e (5-(2-chlorophenyl)-2-methyl-*N*-(m-tolyl)-*5H*-benzo[*h*]chromeno[2,3-*d*]pyrimidin-4-amine)** recorded poor activity.

#### 2.2.2. Five-Dose Assay and Selectivity Estimation

Compound **3a** exhibited broad spectrum antineoplastic efficacy against the nine malignant subpanels examined. A significant cytotoxic effect was observed against all 60 of the human cancer cell lines under examination, with a log_10_ GI_50_ ranging from −4.48 to −5.91 (Figure S1); it is considered to be non-selective (Table S2).

Compound **4a** significantly inhibited all nine cancer subpanels that were evaluated, demonstrating significant cytotoxic action with a log_10_ GI_50_ range of −4.00 to −6.41 on the 60 investigated subpanels (Figure S2), but it is, unfortunately, non-selective (Table S3).

#### 2.2.3. Cytotoxicity/Viability Assay for the Most Active Compounds (**3a** and **4a**) versus Positive Control against MCF7 Cell Line

Assays for cellular viability, which include cytotoxic promising agents, are widely employed to evaluate the impact of potential anti-cancer therapies. The quantity of healthy cells in a population is called cellular viability. Consequently, the quantity of healthy cells, either absolute or relative, in a population is measured using cell viability assays. However, the number of killed cells, either absolute or relative, is measured using cytotoxicity assays [59].

It is critical to determine the number of viable cells that remain at the end of the experiment, regardless of the type of cell-based test being utilized. Numerous assay techniques are available that offer complementary insights on how our synthesized compounds affect cell health, through the measurement of indicators of cell activity, such as DNA synthesis, ATP production, metabolic activity, and the quantity of cells existing or dividing within the population [60].

**Figure 6:**
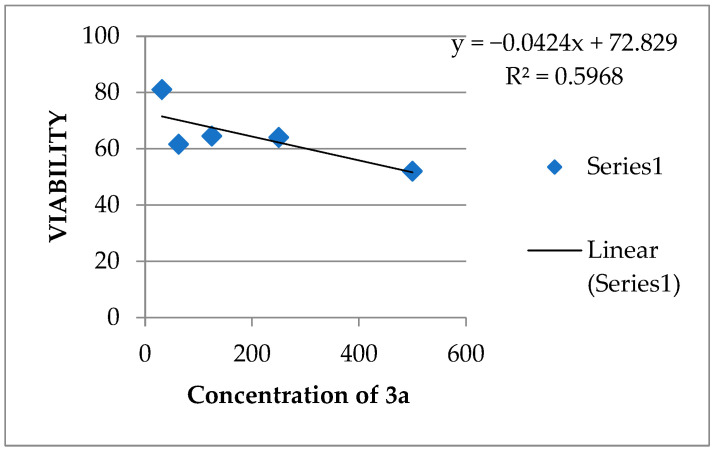

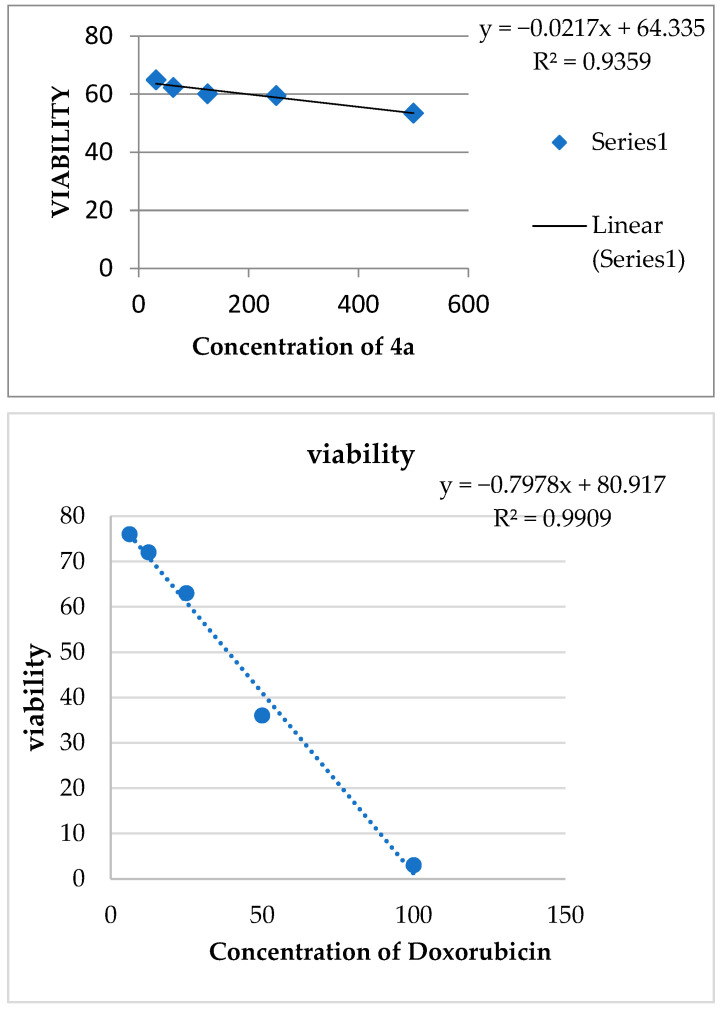
IC_50_ of Compounds **3a** and **4a** and doxorubicin.

From the above-mentioned data and in the light of NCI assay over 60 cell lines, the anti-cancer effect of all newly prepared compounds, the cytotoxicity/viability assay for most active, **4-substitured-5-*para*-methoxyphenyl benzo[*h*]chromeno[2,3-*d*]pyrimidine** (**4-chloro 3a and 4-arylamino 4a**), was evaluated against positive control (**doxorubicin**) [61]. To conclude, the most active compounds (**3a** or **4a**) were the materials evaluated against the human tumor cell line(s), MCF7 (human breast cancer cell line). Using the MTT test, sample concentrations ranged from 500 to 31.25 µg/mL. The IC_50_ was computed (Table 1) and graphs were generated; Figure 6. Based on our data in Table 1, we decided that **3a (4-chloro-5-methoxyphenyl)** was the most effective, **over-all selected compound (NCI-5th dose).**

#### 2.2.4. Cell Cycle Arrest and Apoptotic Cells Formation

**Figure 7:**
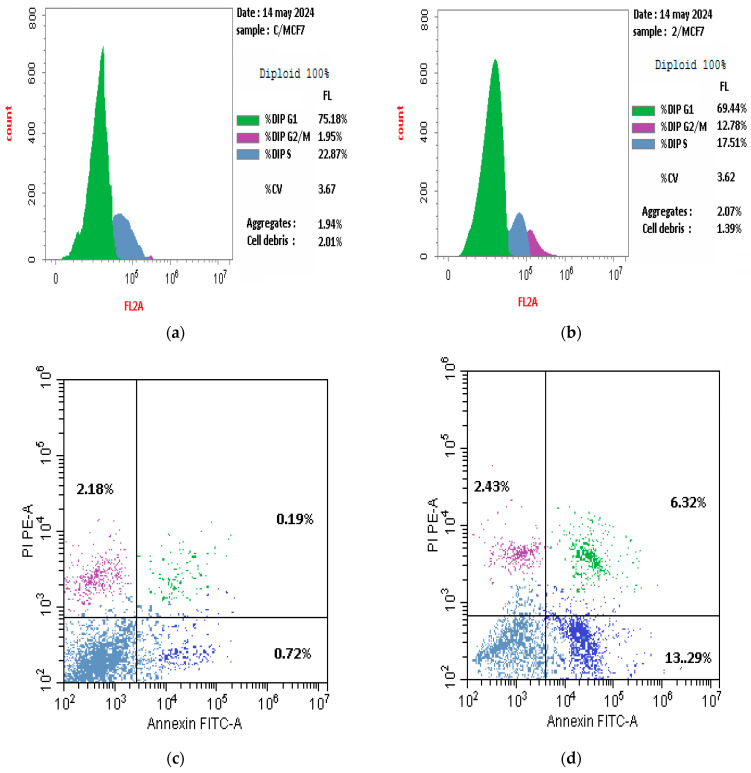
Effect of compound **3a** on cell cycle arrest in MCF-7 cells, (**a**,**c**) represent the control while (**b**,**d**) represent compound **3a.**

The former results indicate that **3a (4-chloro-5-*para*-methoxyphenyl)** was the most effective derivative. Therefore, the current study utilized flow cytometry to determine the dispersion of the cell cycle in the MCF-7 human breast cancer cell line. The majority of MCF7 breast cancer cells, specifically 75.18%, are found in the G1 phase, whereas a smaller proportion, 22.87%, are in the S phase. The majority of cells are located in the pre-G1 phase, whereas a minority of cells are found in the G2/M phases, specifically 1.95%. Conversely, the flow cytometric cell cycle study of breast cancer cell lines showed that 69.44% of cells were found in the G1 phase, while a smaller proportion were in the G2/M phases (12.78%) and the S phase (17.51%). The administration of compound **3a** resulted in the cessation of the cell cycle at the G2/M stages, as demonstrated in Figure 7.

**Figure 8:**
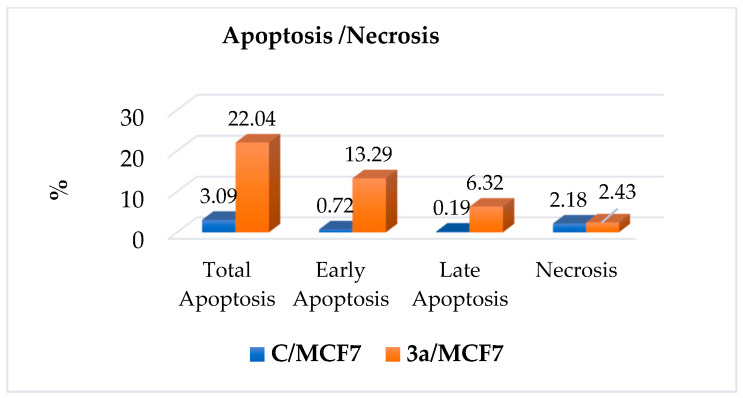
The apoptosis and necrosis of **3a** and control on human MCF-7 breast cancer cells.

The measurement of cellular apoptosis was performed using flow cytometry after 24 h. The breast cancer cell lines investigated revealed an increased % of apoptotic cells (including early, late, and necrotic cells) after treatment with compound **3a**, compared to the control. Figure 8 shows significant increases in the percentage of apoptotic cells in **3a** against control.

#### 2.2.5. RT-PCR

The data presented in Table 2 clearly show the effect of compound **3a** causing downregulation of both antiapoptotic proteins Bcl-2 and Mcl-1 in breast cancer cell line MCF-7 and the significant variations in the expression of the Bcl-2 and Mcl-1 genes. The data demonstrate a notable reduction in the expression of Bcl-2 and Mcl-1 genes in the group treated with compound **3a**, as compared to the control group.

### 2.3. Molecular Docking Analysis

#### 2.3.1. Structure Activity Correlation

Any new Bcl-2/Mcl-1 dual inhibitor must obtain benefits from the canonical interaction mechanism between BH3-only proteins and Bcl-2 proteins, in order to study its structural interaction with Bcl-2/Mcl-1 on the protein level. Bound to the hydrophobic groove of pro-survival Bcl-2 family members, the BH3 domain of the BH3-only protein exhibits binding specificity and selectivity. It is distinguished by four hydrophobic residues and an invariant aspartic acid. This interaction mechanism has led to the development of an anti-cancer treatment that mimics the activity of BH3-only proteins, which bind to and inhibit particular pro-survival Bcl-2 family proteins to cause cancer cells to undergo apoptosis [27,62,63]**.**

**Figure 9:**
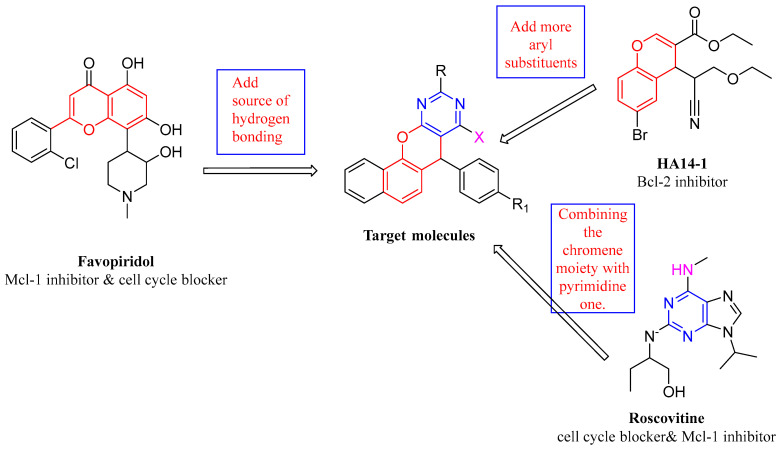
Rational schematic diagram of preparing target compounds.

Here, we attempt to investigate **3a** and **4a** behavior to the hydrophobic common pockets, p4 and p2, based on a previously described Bcl-2/Mcl-1 dual inhibitor, that synchronously occupies the p2 and p4 hydrophobic pockets of Bcl-2 and Mcl-1. A SAR investigation was used to produce novel derivatives that can occupy the p2 and p4 more thoroughly and profoundly than a typical ligand. In specifics, to improve lipophilicity and make our compounds better suited to the p2 pocket, we replaced the 4-bromophenyl in HA 14-1 with a naphthalene ring, fused the pyran ring with a pyrimidine, and, finally, changed the aliphatic substituent with a substituted phenyl. Furthermore, for Mcl-1 inhibitors and cell cycle arrests like rescovitine, as well as flavopiridol, we bioisosterically replaced the chromene and pyrimidine with benzo[*h*]chromeno[2,3-*d*]pyrimidine and modified the nitrogen atom in rescovitine to a chloride atom and/or NH (Figure 9).

The aforementioned SAR study indicates that the benzene ring attached to nitrogen does not extend deeply into the p4 pockets; instead, it floats on the surface of the p4 pocket. To orient the benzene ring into the bottom of the p4 groove, applying the angle between carbon and oxygen, an oxygen or nitrogen atom must be substituted. This could facilitate the formation of more advantageous interactions between the hydrophobic pocket and hydrophobic substituents [27,63].

#### 2.3.2. Molecular Docking Investigation

**Figure 10:**
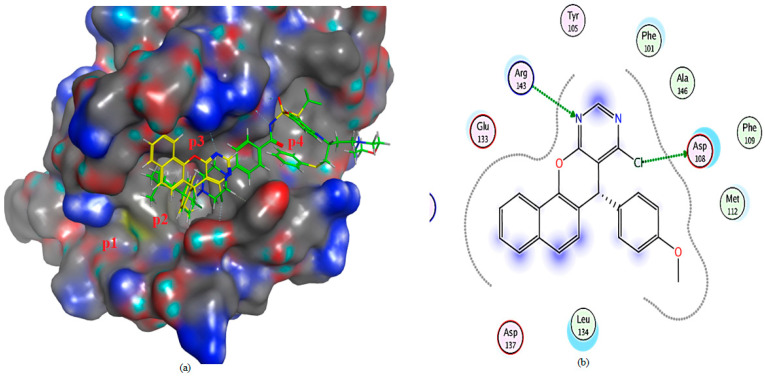
(**a**) The 3D shape of **3a** (yellow color) and **ligand** (green color) on the active site of Bcl-2, (**b**) 2D shape of **3a** on the active site of Bcl-2.

**Figure 11:**
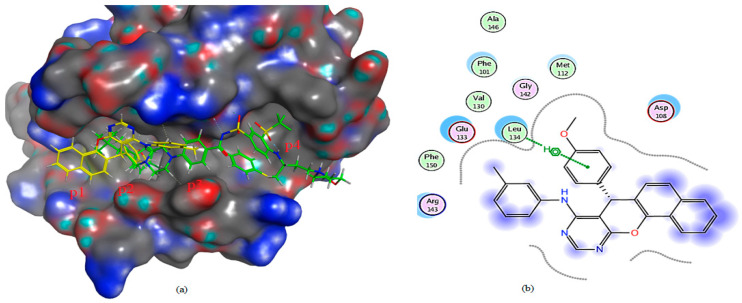
(**a**) The 3D shape of **4a** (yellow color) and **ligand** (green color) on the active site of Bcl-2, (**b**) 2D shape of **4a** on the active site of Bcl-2.

**Figure 12:**
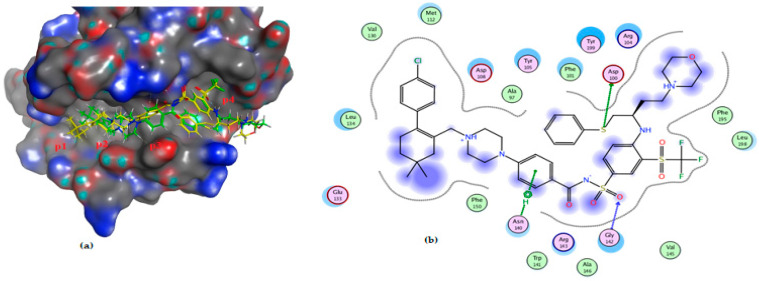
(**a**) The 3D shape of redocked ligand (yellow color) and **ligand** (green color) on the active site of Bcl-2, (**b**) 2D figure of the redocked ligand**,** (**1XJ**), on the active site of bcl2 (PDB: 4LVT).

The molecular docking study of compound **3a** on Bcl-2 reveals that almost the whole compound lies in p2 “the hot spot in both Bcl-2 and Mcl-1” and the p3 pocket; furthermore, it binds through a hydrogen bond with Arg 143; also, the chloride atom formed a hydrogen bond with Asp 108. Compound **4a** lies in P1 and p2 pockets and binds via hydrophobic interaction with leu 134 compared to the ligand, which binds via a hydrogen bond with both Asp 100 and Gly 142, and also through a hydrophobic bond with Asn142 (Table 3 and Figure 10, Figure 11 and Figure 12).

**Figure 13:**
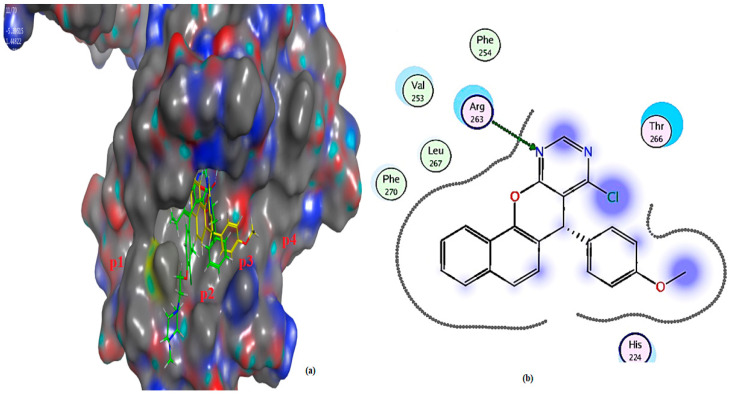
(**a**) The 3D shape of **3a** (yellow color) and **ligand** (green color) on the active site of Mcl-1, (**b**) 2D shape of **3a** on the active site Mcl-1.

**Figure 14:**
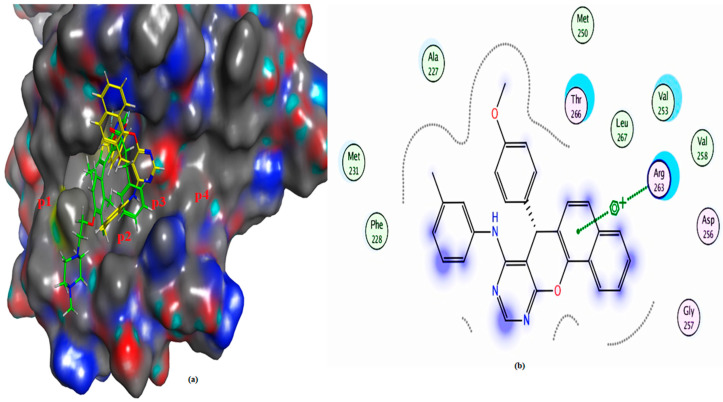
(**a**) The 3D shape of **4a** (yellow color) and **ligand** (green color) on the active site of Mcl-1, (**b**) 2D shape of **4a** on the active site Mcl-1.

**Figure 15:**
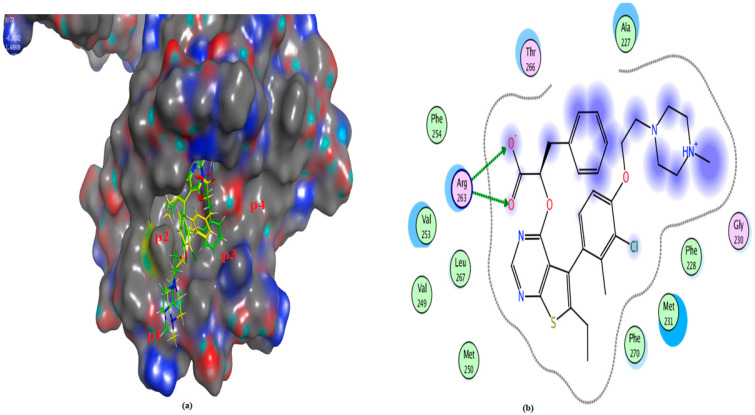
(**a**) The 3D shape of redocked ligand (yellow color) and **ligand** (green color) on the active site of Mcl-1, (**b**) 2D figure of the redocked ligand, (**JLH**), on the active site of Mcl-1 (PDB: 6QYO).

Mcl-1 docking analysis of **3a** shows that it lies in the p2 and p3 pockets, “the hot spots of Mcl-1” protein. The nitrogen atom formed hydrogen bonds with the crucial amino acid in Mcl-1 protein Arg 263. On the other hand, **4a** binds via hydrophobic interaction with key amino acid Arg 263, making it less active, while the ligand binds through two hydrogen bonds with Arg 263 (Table 4 and Figure 13, Figure 14 and Figure 15). The effective binding of chloro substituent 3a and its ability to conserve both aspartic as well as arginine binding, the main feature of both Bcl2 and Mcl1 [27,62,63] besides lipophilic moiety, illustrates the higher efficacy of 3a as an inhibitor of both Bcl2 and Mcl-1 in MCF-7.

## 3. Materials and Methods

### 3.1. Synthesis of Lead Compounds

All of the commercial chemicals that were employed in this study as reagents and starting materials were reagent quality and were acquired from Merck in Darmstadt, Germany. All melting points are measured with an Electro-thermal 1A 9100 instrument (Shimadzu, Kyoto, Japan) and are uncorrected. Using potassium bromide (KBr) discs, infrared spectra (IR) were recorded using a Perkin-Elmer 1650 Spectrophotometer (Waltham, MA, USA). IR was performed in either The Central Laboratory, Faculty of Science, Helwan University, Helwan, Cairo, Egypt, or in the Microanalytical Centre, Faculty of Science, Cairo University, Giza, Egypt, (ref; the attached data). Using tetramethyl silane (TMS) as an internal standard, proton nuclear magnetic resonance (^1^H-NMR) spectra were acquired in dimethyl sulfoxide (DMSO-*d*_6_), on a 300 MHz Varian ^1^H-NMR spectrophotometer. Chemical shifts are represented in (δ) ppm units. The Microanalytical Unit in the Faculty of Pharmacy at Ain-Shams University in Cairo, Egypt or The Microanalytical Centre in the Faculty of Science at Cairo University in Giza, Egypt are the locations where ^1^H-NMR was conducted, (ref; the attached data). El MS-1000 EX (Shimadz, Kyoto, Japan) with electron energy 70 eV was used to perform the mass spectra at the Microanalytical Centre, Faculty of Science, Cairo University, Giza, Egypt. The Microanalytical Center at Cairo University’s Faculty of Science, located in Giza, Egypt, conducted elemental analyses. Using (Merck) Silica gel 60 (particle size 0.06–0.20 mm), column chromatography was carried out. All compounds synthesized in this study are novel and confirmed through their spectral data except compounds **1a**–**c** and **2a**–**e**, which were previously prepared [64,65].

#### 3.1.1. General Procedure for the Synthesis of Compounds **3a**–**e**

A mixture of the appropriate benzo[*h*]chromeno[2,3-*d*] pyrimidines (**2a**–**e**) (0.01mol) was heated under reflux with POCl_3_ (20mL) for 20 h. The reaction mixture was allowed to cool and poured onto ice, the produced precipitate was filtered, dried, and recrystallized from ethanol to give compounds **3a**–**e**.

***4-Chloro-5-(4-methoxyphenyl)-5H-benzo[h]chromeno[2,3-d]pyrimidine (*3a*):*** Yield: 72%; m.p.: 218–220 °C; IR (KBr) υ (cm^−1^): 1606 (C=N), 1157 (C-O); MS (EI) *m*/*z*: 376 (M+2^+^, 5.5%), 374 (M^+^, 14.6%); ^1^H-NMR (DMSO-*d*_6_, 300 MHz) δ (ppm): 3.88 (s, 3H, OCH_3_), 5.16 (s, 1H, C5-H), 6.67–8.47 (m, 11H, Ar-H); Anal. Calcd. for C_22_H_15_ClN_2_O_2_ (374.82): C, 70.50; H, 4.03; N, 7.47%. Found: C, 70.73; H, 4.20; N, 7.57%.

***4-Chloro-5-(2-chlorophenyl)-5H-benzo[h]chromeno[2,3-d]pyrimidine (*3b*):*** Yield: 63%; m.p.: 219–221 °C; IR (KBr) υ (cm^−1^): 1629 (C=N), 1150 (C-O); MS (EI) *m*/*z*: 382 (M+4^+^, 3.3%), 380 (M+2^+^, 20.0%), 378 (M^+^, 30.2%); ^1^H-NMR (DMSO-*d*_6_, 300 MHz) δ (ppm): 5.02 (s, 1H, C5-H), 6.85–8.50 (m, 11H, Ar-H); Anal. Calcd. for C_21_H_12_Cl_2_N_2_O (379.24): C, 66.51; H,3.19; N, 7.39%. Found: C, 66.32; H, 3.14; N, 7.28%.

***4-Chloro-2-methyl-5-phenyl-5H-benzo[h]chromeno[2,3-d]pyrimidine (*3c*):*** Yield: 82%; m.p.: 173–175 °C; IR (KBr) υ (cm^−1^): 1607 (C=N), 1358 (C-O); MS (EI) *m*/*z*: 360 (M+2^+^, 9.4%), 358 (M^+^, 28.2%); ^1^H-NMR (DMSO-*d*_6_, 300 MHz) δ (ppm): 2.60 (s, 3H, CH_3_), 5.60 (s, 1H, C5-H), 7.19–8.31 (m, 11H, Ar-H); Anal. Calcd. for C_22_H_15_ClN_2_O (358.83): C, 73.64; H, 4.21; N, 7.81%. Found: C, 73.58; H, 4.16; N, 7.66%.

***4-Chloro-5-(4-methoxyphenyl)-2-methyl-5H-benzo[h]chromeno[2,3-d]pyrimidine (*3d*):*** Yield: 77%; m.p.: 184–186 °C; IR (KBr) υ (cm^−1^): 1620 (C=N), 1198 (C-O); MS (EI) *m*/*z*:390 (M+2^+^, 8.7%), 388 (M^+^, 24.0%); ^1^H-NMR (DMSO-*d*_6_, 300 MHz) δ (ppm): 2.58 (s, 3H, CH_3_), 3.69 (s, 3H, OCH_3_), 5.51 (s, 1H, C5-H), 6.79–8.44 (m, 10H, Ar-H); Anal. Calcd. for C_23_H_17_ClN_2_O_2_ (388.85): C, 71.04; H, 4.41; N, 7.20%. Found: C, 71.13; H, 4.43; N, 7.29%.

***4-Chloro-5-(2-chlorophenyl)-2-methyl-5H-benzo[h]chromeno[2,3-d]pyrimidine (*3e*):*** Yield: 75%; m.p.: 228–230 °C; IR (KBr) υ (cm^−1^): 1603 (C=N), 1369 (C-O); MS (EI) *m*/*z*: 396 (M+4^+^, 3.4%), 394 (M+2^+^, 10.9%), 392 (M^+^, 16.3%); ^1^H-NMR (DMSO-*d*_6_, 300 MHz) δ (ppm): 2.58 (s, 3H, CH_3_), 5.95 (s, 1H, C5-H), 7.23–8.28 (m, 10H, Ar-H); Anal. Calcd. for C_22_H_14_Cl_2_N_2_O (393.27): C, 67.19; H, 3.59; N, 7.12%. Found: C, 67.35; H, 3.70; N, 7.23%.

#### 3.1.2. General Procedure for the Synthesis of Compounds **4a**–**e**

A mixture of 4-chloro-pyrimidines (**3a**–**e**) (0.01 mol) was separately heated under reflux with *m*-toluidine (2.14g, 0.02 mol) in methanol (30 mL) containing a few drops of pyridine for 8–12 h. The reaction mixture was allowed to cool and poured onto ice/water-containing HCl, the produced precipitate was filtered, dried, and recrystallized from acetic acid to give compounds **4a**–**e.**

***5-(4-Methoxyphenyl)-N-(m-tolyl)-5H-benzo[h]chromeno[2,3-d]pyrimidin-4-amine (*4a*):*** Yield: 59%; m.p.: 201–203 °C; IR (KBr) υ (cm^−1^): 3432 (NH), 1606 (C=N), 1220 (C-O); MS (EI) *m*/*z*: 445 (M^+^, 42.6%); ^1^H-NMR (DMSO-*d*_6_, 300 MHz) δ (ppm): 3.68 (s, 3H, C3^−^-CH_3_), 3.88 (s, 3H, OCH_3_), 5.17 (s, 1H, C5-H), 6.75–8.0 (m, 15H, Ar-H), 8.42 (s, 1H, NH, D_2_O exchangeable); Anal. Calcd. for C_29_H_23_N_3_O_2_ (445.52): C, 78.18; H, 5.20; N, 9.43%. Found: C, 78.26; H, 5.13; N, 9.71%.

***5-(2-Chlorophenyl)-N-(m-tolyl)-5H-benzo[h]chromeno[2,3-d]pyrimidin-4-amine (*4b*):*** Yield: 54%; m.p.: 131–133 °C; IR (KBr) υ (cm^−1^): 3416 (NH), 1626 (C=N), 1220 (C-O); MS (EI) *m*/*z*: 451 (M+2^+^, 9.1%), 449 (M^+^, 27.3%); ^1^H-NMR (DMSO-*d*_6_, 300 MHz) δ (ppm): 3.49 (s, 3H, C3^−^-CH_3_), 5.70 (s, 1H, C5-H), 6.96–7.85 (m, 15H, Ar-H), 8.56 (s, 1H, NH, D_2_O exchangeable); Anal. Calcd. for C_28_H_20_ClN_3_O (449.94): C, 74.75; H, 4.48; N, 9.34%. Found: C, 74.48; H, 4.49; N, 9.53%.

***2-Methyl-5-phenyl-N-(m-tolyl)-5H-benzo[h]chromeno[2,3-d]pyrimidin-4-amine (*4c*):*** Yield: 69%; m.p.: 141–143 °C; IR (KBr) υ (cm^−1^): 3400 (NH), 1620 (C=N), 1358 (C-O); MS (EI) *m*/*z*: 429 (M^+^, 37.7%); ^1^H-NMR (DMSO-*d*_6_, 300 MHz) δ (ppm): 2.71 (s, 3H, C3^−^-CH_3_), 2.87 (s, 3H, C2-CH_3_), 5.62 (s, 1H, C5-H), 7.27–8.28 (m, 15H, Ar-H), 8.39 (s, 1H, NH, D_2_O exchangeable); Anal. Calcd. for C_29_H_23_N_3_O (429.52): C, 81.09; H, 5.40; N, 9.78%. Found: C, 81.03; H, 5.51; N, 9.71%.

***5-(4-Methoxyphenyl)-2-methyl-N-(m-tolyl)-5H-benzo[h]chromeno[2,3-d]pyrimidin-4-amine (*4d*):*** Yield: 73%; m.p.: >300 °C; IR (KBr) υ (cm^−1^): 3328 (NH), 1606 (C=N), 1254 (C-O); MS (EI) 459 (M^+^, 16.9%); ^1^H-NMR (DMSO-*d*_6_, 300 MHz) δ (ppm): 2.60 (s, 3H, C3^−^-CH_3_), 3.66 (s, 3H, C2-CH_3_), 3.91 (s, 3H, OCH_3_), 5.52 (s, 1H, C5-H), 6.83–8.42 (m, 14H, Ar-H), 8.47 (s, 1H, NH, D_2_O exchangeable); Anal. Calcd. for C_30_H_25_N_3_O_2_ (459.55): C, 78.41; H, 5.48; N, 9.14%. Found: C, 78.17; H, 5.34; N, 9.21%.

***5-(2-Chlorophenyl)-2-methyl-N-(m-tolyl)-5H-benzo[h]chromeno[2,3-d]pyrimidin-4-amine (*4e*):*** Yield: 78%; m.p.: 233–235 °C; IR (KBr) υ (cm^−1^): 3390 (NH), 1605 (C=N), 1370 (C-O); MS (EI) *m*/*z*: 465 (M+2^+^, 13.5%), 463 (M^+^, 40.6%); ^1^H-NMR (DMSO-*d*_6_, 300 MHz) δ (ppm): 2.44 (s, 3H, C3^−^-CH_3_), 2.61 (s, 3H, C2-CH_3_), 6.01 (s, 1H, C5-H), 7.23–7.92 (m, 14H, Ar-H) 8.31 (s, 1H, NH, D_2_O exchangeable); Anal. Calcd. for C_29_H_22_ClN_3_O (463.97): C, 75.07; H, 4.78; N, 9.06%. Found: C, 75.21; H, 4.57; N, 8.83%.

### 3.2. Assessment of Anti-Cancer Activity

#### 3.2.1. The NCI-60 Human Tumor Cell Lines Screen

This screen employs 60 distinct human tumor cell lines (the cancer cell lines MCF-7 was purchased from the American Type Culture Collection (ATCC), located in Manassas, VA, USA. Tissue culture media was obtained from Invitrogen-Life Technologies. MTT was purchased from Acros Organics™ Thermo Fisher Scientific, Waltham, MA, USA. Doxorubicin (DOX) was obtained from the drugstore in Egypt) derived from leukemia, melanoma, and cancers of the colon, lung, brain, kidney, ovary, prostate, and breast.

##### Selection Guidelines in NCI

Structures are often chosen for screening based on their capacity to provide diversity to the NCI small molecule chemical library (in compliance with the NCI’s Drug Evaluation Branch’s protocol, Bethesda, Rockville, MD, USA) [66,67].

The NCI selected all the newly prepared benzo[*h*]chromeno[2,3-*d*]pyrimidine derivatives for single-dose testing (10^−5^ M). NCI 60 single-dose testing is performed in all 60 cell lines according to the reported assay [67,68]. Compounds were dissolved in DMSO: glycerol (9:1) and are stored in a −70 °C freezer. The prepared compounds were added at single concentration of 10^−5^ M and the culture was incubated for 48 h. End-point determinations were made with a protein-binding dye, sulforhodamine B (SRB). The human cancerous cell lines were derived from nine different cancer types: leukemia, melanoma, CNS, lung, colon, ovarian, prostate, renal, and breast cancers.

##### Interpretation of One-Dose Data

The one-dose data are reported as a mean of a graph of the percent growth of treated cells. The number reported for the one-dose assay is growth relative to the no-drug control, and relative to the time zero number of cells. This allows for detection of both growth inhibition (values between 0 and 100) and lethality (values less than 0). As for example, a value of 20 means 80% growth inhibition, a value of 100 would mean there is no inhibition, and a value of −60 represents 60% lethality [68].

##### NCI-60 Cell Five-Dose Screen

Compounds that exhibit substantial growth inhibition in the one-dose Screen (eight or more cell lines with a growth % of 10 or less), such as compounds **3a** and **4a**, are tested against the 60 cell panels at five concentration levels. The compounds’ growth inhibitory and/or cytotoxic activity in this testing was examined against a panel of 60 human tumor cell lines derived from nine different types of cancer at a 10-fold dilution of five doses ranging from 10^−4^ to 10^−8^ M. At the end of the 48 h incubation period, three dosage response parameters “GI_50_ (Median Growth Inhibitory Concentration), TGI (Total Growth Inhibitory Concentration), and LC_50_ (Median Lethal Concentration)” were determined [66,67,68,69] for each cell line of **3a** and **4a**. Together with the range and delta parameters, the average activity parameter (MG_MID) for all cell lines was also ascertained. Next, the means of the log_10_, s of each unique GI_50_, TGI, and LC_50_ value, which are the log_10_ GI_50_, log_10_ TGI, and log_10_ LC_50,_ were determined. Negative numbers represent the most sensitive cell lines. Compounds with log_10_ GI_50_ values ≤ −4 are considered active. Moreover, by dividing the entire panel MID^a^ by each subpanel MID^b^ (µM), we obtained the ratio that is used to calculate the selectivity of the molecule, where MID^a^ is the test agent’s average (µM) sensitivity across all cell lines and MID^b^ is the average of cell lines in each subpanel. Ratios > 6 indicate increased selectivity for the related cell line, whereas ratios between 3 and 6 indicate moderate selectivity. Compounds that do not fit any of these criteria are considered to be non-selective [70].

#### 3.2.2. Cell Viability and Cytotoxicity against MCF-7 Cell Line

The assessment of cell viability was conducted by measuring the reduction of yellow MTT (3-(4,5-dimethylthiazol-2-yl)-2,5-diphenyl tetrazolium bromide) to purple formazan, a process that is dependent on the presence of mitochondria [71].

***Procedure:*** The aforementioned procedures were conducted in a sterile environment utilizing a Laminar flow biosafety cabinet Class II A2, manufactured by Labconco (Kansas City, MO, USA). The cells were placed in a suspension of DMEM media with 1% antibiotic–antimycotic mixture (10,000 U/mL potassium penicillin, 10,000 µg/mL streptomycin sulfate, and 25 µg/mL amphotericin B), 1% L-glutamine, and 5% fetal bovine serum. The suspension was maintained at a temperature of 37 °C under 5% CO_2_ using a CO_2_ incubator (Sartorius stedium, biotech, Goettingen, Germany). The cells were cultured in batches for a period of 10 days. After that, they were placed in 96-well plastic plates at a concentration of 10 × 10^3^ cells per well, in fresh complete growth medium. The plates were then kept at a temperature of 37 °C for 24 h, with a CO_2_ concentration of 5%. The cells were either left alone (as a negative control) or treated with various concentrations of drugs, resulting in final concentrations of 500, 250, 125, 62.5, and 31.25 µg/mL. After incubating for 48 h, the medium was removed and 20 microliters of MTT salt (2.5 µg/mL) were added to each well. The cells were then incubated for an additional 4 h at 37 °C with 5% carbon dioxide. In order to halt the reaction and dissolve the crystals that had formed, a solution containing 200 μL of 10% sodium dodecyl sulphate (SDS) in 0.01M HCl was introduced into each well. The mixture was then incubated at a temperature of 37 °C for the duration of one night. A positive control (doxorubicin) consisting of a concentration of 100 µg/mL was utilized as a well-known cytotoxic substance that induces 100% fatality under identical conditions [72,73].

The measurement of absorbance was conducted using a microplate multi-well reader (Bio-Rad Laboratories Inc., model 3350, Hercules, CA, USA) at a wavelength of 595 nm, with a reference wavelength of 620nm. The IC_50_ values were computed, and graphs were generated using SPSS one-way ANOVA. All experiments were conducted in triplicate.

Viability = absorbance of drug/absorbance of control × 100

Cytotoxicity = 100 − viability

#### 3.2.3. Cell Cycle and Apoptosis Detection by Flow Cytometry

After 24 h of incubation, MCF-7 cells (3 × 10^5^ cells/well) were harvested with trypsinization from all groups, either treated or untreated, and washed twice in ice-cold PBS and fixed with 70% ethanol at 4 °C overnight. Cells were then washed in PBS, centrifuged, and stained with propidium iodide (PI) (CAT# ab139418) (50 µg/mL) for cell cycle analysis. Staining was assessed in a FACSC onto II flow cytometer, followed by analysis using BD Accuri-C6 Plus software (Biosciences, CA, USA) [74].

#### 3.2.4. Gene Expression Analysis of Bcl2 and Mcl1 by Reverse Transcription-Quantitative Polymerase Chain Reaction (RT-qPCR)

RNA isolation and reverse transcription: RNA was extracted from breast cancer cells (MCF-7) using the RNase plus mini kit (Qiagen, Venlo, The Netherlands), following the manufacturer’s instructions. The kit included a DNaseon-column treatment that removed genomic DNA. The RNA concentration was quantified spectrophotometrically at 260 nm using the Nano Drop ND-1000 spectrophotometer (Thermo Fisher Scientific, Waltham, MA, USA), and the absorbance ratio at 260/280 nm was used to confirm RNA purity. RNA integrity was evaluated using formaldehyde agarose gel electrophoresis on 2% agarose gels. RNA (1 µg) was utilized to synthesize cDNA using the Reverse Transcription System (Promega, Leiden, The Netherlands). To prevent secondary formations, whole RNA was incubated at 70 °C for 10 min. RNA was treated with MgCl_2_ (25 mM), RTase buffer (10×), dNTP mixture (10 mM), oligod (t) primers, RNase inhibitor (20 U), and AMV reverse transcriptase (20 U/µL). This mixture was incubated at 42 °C for one hour. qRT-PCR was carried out on an optical 96-well plate using an ABI PRISM7500 quick sequence detection system (Applied Biosystems, Carlsbad, CA, USA) with universal cycling conditions of 40 cycles of 15 s at 95 °C and 60 s at 60 °C following a 10 min denaturation step at 95 °C. To each 10 µL reaction, we added 5 µL of SYBR Green Master Mix (Applied Biosystem), 0.3 µL of gene-specific forward and reverse primers (10 µM), 2.5 µL of cDNA, and 1.9 µL of nuclease-free water. Table 5 shows the sequences of the PCR primer pairs utilized for each gene. All data were adjusted to the endogenous control GAPDH.

### 3.3. Docking Studies

Molecular Operating Environment (MOE) software (version 2019.01) was used to dock our most active derivatives (**3a** and **4a**) into the 3D forms of both Bcl-2 and Mcl-1 with the proper ligands: the crystal structures of Bcl-2 (PDB: 4LVT) at 2.6 Å resolution and the crystal structure of Mcl-1 (PDB code: 6QYO) at 2.6 Å resolution. As a representation of the conformational configurations with the most favorable binding energy, the predicted binding of the target derivatives to each Bcl-2 and Mcl-1 active pocket was found to be the best categorized scoring function (ΔE).

## 4. Conclusions

**Figure 16:**
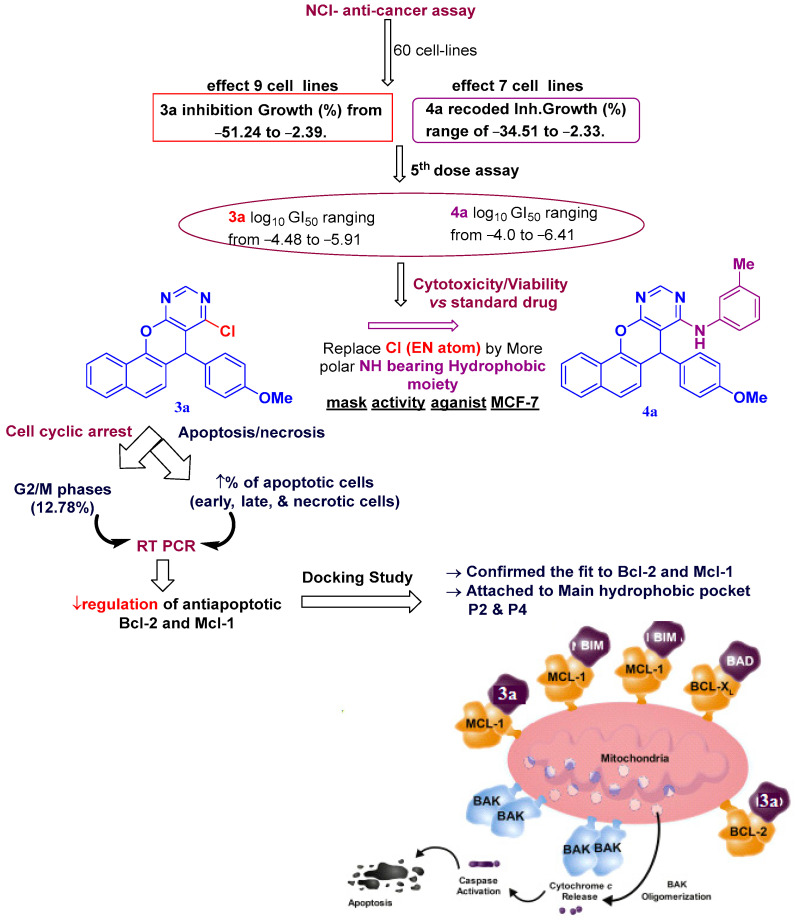
SAR for most active chromenopyrimine (**3a** and **4a**).

The abovementioned results indicate that, in addition to their crucial roles as agents of cell cycle arrest and apoptosis induction, the intriguing discovery about the interactions between the structures of chromenopyrimidines and the Bcl-2 and Mcl-1 proteins/antagonist highlights the promising effect of **3a** and **4a** as anti-breast cancer agents. Conclusively, **3a** was chosen as a new fused chromenopyrimidine with dual inhibitory activity against Bcl-2 and Mcl-1, potentially causing cell cycle arrest or apoptosis. Figure 16 presents the findings.

## Figures and Tables

**Table 1:** The half-maximal inhibitory (IC_50_) concentration of our compounds.

Cytotoxicity of Most Active Derivatives (5-Dose Active) against Standard **										
Tested Compound	3a (4-Chloro)					4a (4-Tolylamino)				
	Different Dilution (µg/mL)					Different Dilution (µg/mL)				
	500	250	125	62.5	31.25	500	250	125	62.5	31.25
Viability %	52 ± 1.22	64 ± 1.16	64.5 ± 1.22	61.5 ± 1.35	81 ± 1.70	53.5 ± 0.44	59.5 ± 0.79	60.5 ± 0.80	62.37 ± 0.59	65 ± 0.43
Cytotoxicity %	48 ± 1.22	36 ± 1.16	35.5 ± 1.22	38.4 ± 1.35	19 ± 1.70	46.5 ± 0.44	40.4 ± 0.79	39.4 ± 0.80	37.63 ± 0.59	35 ± 0.43
* IC_50_ µg/ml	399.6891					631.2369				

* IC_50_: Lethal concentration of the sample that causes the death of 50% of cells in 48 h. ** Doxorubicin (different dilution according to standard methods is IC_50_ (=38.75 µg/mL) [61].

**Table 2:** The Bcl-2 and Mcl-1 gene expression mRNA fold change measured by RT-qPCR in various groups.

Groups	Bcl-2	Mcl-1
Control	1 ± 0.00	1 ± 0.00
Compound **3a**	0.77 ± 0.05 *	0.71 ± 0.04 *

* Mean SE is the statistical distribution used to represent values. A *p*-value < 0.05 was interpreted as demonstrating statistical significance. Significant when compared to control at *p* < 0.05.

**Table 3:** Molecular docking results of molecule **3a** and **4a** versus reference (1XJ) in Bcl-2 active site (PDB: 4LVT).

Compound	Docking Score (S) Kcal/mol	RMSD	E-Score 1 (London dG) Kcal/mol	E-Score 2 (London dG) Kcal/mol	Binding Interaction (Ligand Receptor)
**3a**	−5.5000	2.0588	−8.1277	−5.5000	(N-Arg143) (H-b) (Cl-Asp108) (H-b)
**4a**	−5.67806	1.65981	−8.56915	−5.67806	(benzene H-Leu 134) (pi-H)
Receptor ligand, (1XJ)	−8.7886	2.0588	−8.8739	−8.7886	(benzene H-Asn 140) (pi-H) (S-Asp 100) (H-b) (Gly 142-O) (H-b)

**Table 4:** Molecular docking results of **3a** and **4a** versus reference (JLH) in Mcl-1active site (PDB: 6QYO).

Compound	Docking Score (S) Kcal/mol	RMSD	E-Score 1 (London dG) Kcal/mol	E-Score 2 (London dG) Kcal/mol	Binding Interaction (Ligand Receptor)
**3a**	−5.3952	1.4482	−8.7543	−5.3952	(N-Arg 263) (H-b)
**4a**	−5.9229	1.7320	−9.7526	−5.9229	(benzene + Arg363) (Pi- +)
Receptor ligand, (JLH)	−6.9102	1.4881	−9.9729	−8.7886	(Arg 263-O) (H-b) (Arg 263-O) (H-b)

**Table 5:** Bcl-2, Mcl-1, and GAPDH gene primer sequences.

Target	Sequence
**Bcl-2**	F 5′-ATCGCCCTGTGGATGACTGAGT-3′ R 5′-GCCAGGAGAAATCAAACAGAGGC-3′
**Mcl-1**	F 5′-CCAAGAAAGCTGCATCGAACCAT-3′ R 5′-CAGCACATTCCTGATGCCACCT-3′
**GAPDH**	F 5′-GTCTCCTCTGACTTCAACAGCG-3′ R 5′-ACCACCCTGTTGCTGTAGCCAA-3′

## Data Availability

Data are contained within the article and Supplementary Materials.

## Supplementary Materials

The following supporting information can be downloaded at: https://www.mdpi.com/article/10.3390/molecules29194697/s1, Figures S1 and S2. Dose response curves of compounds **3a** and **4a** against 60 cancer cell lines; Figures S3–S23. Spectral data of **3a–e: 4a–e**. Table S1. Anticancer testing results for compounds **3a to 4e** (growth percent against 60 cell lines) Table S2. Selectivity of compound **3a** on nine human cancer cell types. Table S3. Selectivity of compound **4a** on nine human cancer cell types.
