# Supplementary material for: Synthesis, Anti-Cancer Activity, Cell Cycle Arrest, Apoptosis Induction, and Docking Study of Fused Benzo[h]chromeno[2,3-d]pyrimidine on Human Breast Cancer Cell Line MCF-7
Source: Molecules. 2024 Oct 4;29(19):4697. doi: 10.3390/molecules29194697 (PMC11478142; doi:10.3390/molecules29194697)
Supplement: Supplementary file 1 [file molecules-29-04697-s001.zip › molecules-3208032-supplementary.pdf]

Table S1: Anticancer testing results for compounds **3a to 4e** (growth percent against 60 cell lines).

**Panel/Cell Line**	**Growth Percent**									
	**3a**	**3b**	**3c**	**3d**	**3e**	**4a**	**4b**	**4c**	**4d**	**4e**
**Leukemia:**										
CCRF-CEM	23.65	62.65	53.29	73.37	104.66	10.98	35.47	60.31	80.60	85.70
HL-60(TB)	21.67	22.32	2.27	26.35	87.51	14.06	57.78	44.28	93.12	95.36
K-562	10.99	23.04	13.82	17.75	79.80	17.72	22.77	12.52	39.52	80.33
MOLT-4	27.04	66.13	28.60	34.80	72.17	25.41	38.22	28.51	58.19	65.09
RPMI-8226	13.06	67.25	43.28	51.50	79.14	25.36	57.98	55.32	71.47	72.48
SR	25.79	36.04	20.88	27.74	98.54	16.66	48.41	13.42	53.75	90.95
**Non-Small Cell Lung Cancer:**										
A549/ATCC	45.94	92.93	62.78	77.79	89.41	42.53	80.67	89.60	93.07	95.43
EKVX	39.67	85.19	74.28	73.86	87.27	36.79	80.04	77.12	83.63	93.73
HOP-62	32.54	77.78	68.36	71.84	89.19	35.38	76.75	66.83	70.60	93.98
HOP-92	70.89	95.41	91.22	77.13	81.32	34.57	91.28	75.27	71.53	79.69
NCI-H226	32.43	90.00	69.74	90.98	78.76	51.70	84.32	79.04	92.24	99.08
NCI-H23	36.71	81.33	66.03	67.08	94.31	29.29	72.93	59.30	68.74	85.54
NCI-H322M	48.02	92.56	89.19	83.32	87.54	56.93	84.88	93.84	93.32	98.13
NCI-H460	11.31	79.68	19.37	57.37	88.39	33.05	72.04	67.42	95.85	98.58
NCI-H522	**-21.71**	4.44	**-7.35**	13.50	78.91	**-16.94**	18.47	29.26	70.72	91.71
**Colon Cancer:**										
COLO 205	34.87	114.91	91.90	105.33	109.42	35.01	91.46	80.11	98.36	107.56
HCC-2998	43.49	81.39	70.33	82.68	87.94	48.44	89.60	75.10	93.20	103.29
HCT-116	18.06	56.23	24.24	48.00	77.32	13.58	58.45	47.38	64.15	87.43
HCT-15	9.17	41.07	24.46	38.48	88.42	24.50	46.88	32.46	54.98	90.60
HT29	14.93	94.68	47.81	86.87	93.14	30.88	82.93	60.06	74.31	85.94
KM12	18.98	57.93	33.08	49.33	95.38	25.78	53.43	55.05	67.19	97.78
SW-620	19.90	52.25	20.72	40.62	94.96	22.59	53.85	38.69	73.05	95.81
**CNS Cancer:**										
SF-268	52.21	89.61	68.93	75.25	102.06	56.85	71.21	89.96	95.50	113.90
SF-295	10.37	67.01	42.00	72.26	76.20	15.32	50.30	84.48	61.44	79.23
SF-539	**-41.08**	80.54	46.83	71.70	83.99	**-11.06**	78.55	80.45	84.76	100.53
SNB-19	32.30	82.61	65.83	78.57	72.95	26.75	78.12	78.32	83.28	92.98
SNB-75	**-21.35**	44.52	23.50	48.78	51.69	51.57	46.06	70.01	47.86	122.06

U251	23.12	76.13	39.19	73.65	97.96	19.57	75.75	75.35	93.06	101.98
**Melanoma:**										
LOX IMVI	20.74	60.75	37.90	45.88	83.64	22.36	69.14	46.31	62.76	92.03
MALME-3M	37.60	65.13	48.07	63.06	93.03	43.95	64.06	56.76	89.38	104.55
M14	13.36	53.84	23.61	49.16	96.03	1.00	60.99	51.20	75.06	83.19
MDA-MB-435	**-51.24**	**-37.42**	**-22.20**	**-13.44**	97.15	**-34.51**	**-40.67**	**-7.61**	18.30	99.73
SK-MEL-2	25.98	57.38	35.32	61.73	106.32	28.66	100.95	67.84	87.55	97.90
SK-MEL-28	52.45	67.05	56.15	62.70	100.36	52.41	66.74	76.66	98.05	125.98
SK-MEL-5	**-7.99**	48.15	32.70	52.66	98.95	**-25.87**	43.24	43.98	86.92	96.18
UACC-257	57.13	91.16	84.50	89.59	107.26	53.15	84.09	90.96	95.91	105.27
UACC-62	22.01	34.37	24.73	39.55	78.21	21.71	34.05	45.93	74.15	77.33
**Ovarian Cancer:**										
IGROV1	33.62	73.44	32.43	46.59	81.15	34.88	72.02	50.94	62.37	89.04
OVCAR-3	**-2.39**	81.49	23.76	75.59	112.63	**-13.18**	57.58	53.42	84.47	114.14
OVCAR-4	68.10	79.94	75.83	77.46	85.62	51.47	70.53	75.56	84.19	96.00
OVCAR-5	45.60	101.86	76.40	83.27	87.66	53.16	98.55	98.33	109.46	107.78
OVCAR-8	33.17	87.13	67.20	86.49	95.32	28.10	74.77	80.37	90.41	96.16
NCI/ADR-RES	**-16.61**	35.00	6.08	18.16	81.64	4.72	43.46	20.51	60.03	87.44
SK-OV-3	35.84	83.04	76.80	79.71	93.95	34.29	65.25	69.16	72.30	80.76
**Renal Cancer:**										
786-0	31.07	90.78	72.54	88.76	101.87	40.21	90.62	92.08	94.48	102.45
A498	67.22	89.66	110.98	108.78	132.55	87.68	102.15	119.62	112.21	117.20
ACHN	32.29	89.95	65.06	74.48	75.30	39.40	80.75	68.34	75.81	88.06
CAKI-1	31.09	49.81	35.57	35.57	50.18	23.31	48.05	36.48	50.69	68.69
RXF 393	**-3.29**	78.99	60.35	71.07	100.17	**-2.33**	71.42	76.57	87.50	102.87
SN12C	32.47	87.53	56.20	79.93	89.31	30.85	76.81	93.67	90.67	102.84
TK-10	120.99	138.46	126.96	169.31	132.63	89.62	114.39	122.67	123.25	114.31
UO-31	28.64	62.77	46.84	44.75	75.26	30.10	64.68	59.17	65.50	75.77
**Prostate Cancer:**										
PC-3	30.12	66.48	54.06	57.52	68.28	24.74	61.73	63.45	71.69	77.33
DU-145	66.48	121.16	100.65	101.54	104.75	41.20	97.91	97.74	81.78	104.89
**Breast cancer:**										
MCF7	5.27	32.33	15.59	16.24	76.83	25.59	55.40	25.26	42.79	75.92
MDA-MB-231/ATCC	15.62	81.02	49.93	43.34	82.48	17.20	52.57	60.01	66.16	86.84
HS 578T	1.98	58.67	46.18	67.84	78.70	9.08	60.36	72.50	79.25	72.63

BT-549	24.06	62.08	39.84	52.87	105.84	1.27	77.15	72.98	111.27	103.36
T-47D	34.62	82.86	55.92	73.79	88.15	33.24	74.97	72.69	75.95	67.51
MDA-MB-468	**-18.24**	35.54	21.54	**-2.12**	78.05	**-27.91**	**-13.09**	76.84	88.23	90.66
**Mean**	25.01	68.77	48.87	61.96	89.46	26.55	64.65	64.16	78.27	93.49
**Delta**	76.25	106.19	71.07	75.40	39.28	61.06	105.32	71.77	59.97	28.40
**Range**	172.23	175.88	149.16	182.75	82.45	124.13	155.06	130.28	104.95	60.89

Table S2: Selectivity of compound **3a** on nine human cancer cell types.

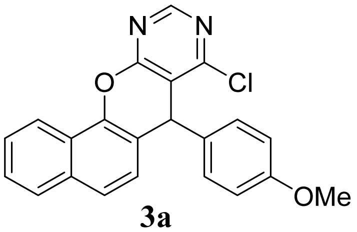

3a

**Panel**	**Cell line**	**GI_{50}** Conc. Per cell line	Subpanel MID[b]	Selectivity ratio	**TGI**	**LC_{50}**
**Leukemia**	CCRF-CEM	3.38	3.55	1.65	>100	>100
	HL-60(TB)	2.70			>100	>100
	K-562	3.53			>100	>100
	MOLT-4	3.14			>100	>100
	RPMI-8226	5.48			>100	>100
	SR	3.05			81.70	>100
**Non-small cell lung cancer**	A549/ATCC	8.03	7.48	0.78	>100	>100
	EKVX	17.20			>100	>100
	HOP-62	4.06			>100	>100
	HOP-92	11.40			91.90	>100
	NCI-H226	6.66			52.30	>100
	NCI-H23	5.05			>100	>100
	NCI-H322M	9.70			>100	>100
	NCI-H460	3.52			18.30	>100
	NCI-H522	1.71			5.63	>100
**Colon cancer**	COLO 205	6.93	5.07	1.15	37.70	>100
	HCC-2998	10.60			33.20	>100
	HCT-116	4.05			>100	>100
	HCT-15	3.38			20.40	>100
	HT29	3.23			18.70	>100

	KM12	3.84			21.60	>100
	SW-620	3.44			>100	>100
**CNS cancer**	SF-268	6.02	3.87	1.51	>100	>100
	SF-295	3.99			50.10	>100
	SF-539	2.65			9.31	71.30
	SNB-19	3.95			>100	>100
	SNB-75	2.70			8.54	>100
	U251	3.90			31.50	>100
**Melanoma**	LOX IMVI	4.22	4.55	1.29	21.10	83.70
	MALME-3M	3.64			>100	>100
	M14	5.03			>100	>100
	MDA-MB-435	1.23			3.35	>100
	SK-MEL-2	3.52			>100	>100
	SK-MEL-28	7.43			>100	>100
	SK-MEL-5	4.16			22.20	>100
	UACC-257	7.37			>100	>100
	UACC-62	4.33			>100	>100
**Ovarian cancer**	IGROV1	4.87	6.00	0.98	>100	>100
	OVCAR-3	3.14			8.89	>100
	OVCAR-4	9.85			>100	>100
	OVCAR-5	11.40			>100	>100
	OVCAR-8	4.87			31.80	>100
	NCI/ADR-RES	2.71			11.90	>100
	SK-OV-3	5.16			>100	>100
**Renal cancer**	786-0	12.50	10.58	0.55	>100	>100
	A498	19.30			>100	>100
	ACHN	6.51			>100	>100
	CAKI-1	3.46			>100	>100
	RXF 393	3.30			28.90	>100
	SN12C	5.03			>100	>100
	TK-10	32.80			>100	>100
	UO-31	1.71			57.20	>100
**Prostate cancer**	PC-3	6.68	6.76	0.87	>100	>100
	DU-145	6.83			>100	>100
	MCF7	3.52	3.77	1.55	83.70	>100

**Breast cancer**	MDA-MB-231/ATCC	4.74			31.10	>100
	HS 578T	2.07			7.37	>100
	BT-549	4.23			>100	>100
	T-47D	5.23			>100	>100
	MDA-MB-468	2.82			8.05	>100
**Mid** [a]	5.85					

Table S3: Selectivity of compound **4a** on nine human cancer cell types.

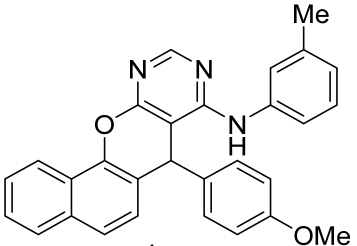

4a

**Panel**	**Cell line**	**GI_{50}**			**TGI**	**LC_{50}**
		Conc. Per cell line	Subpanel MID[b]	Selectivity ratio		
**Leukemia**	CCRF-CEM	3.00	2.25	2.61	>100	>100
	HL-60(TB)	2.01			6.48	>100
	K-562	1.58			>100	>100
	MOLT-4	2.49			>100	>100
	RPMI-8226	3.23			>100	>100
	SR	1.18			>100	>100
**Non-small cell lung cancer**	A549/ATCC	4.29	4.10	1.43	>100	>100
	EKVX	4.16			>100	>100
	HOP-62	2.71			>100	>100
	HOP-92	3.81			>100	>100
	NCI-H226	4.47			>100	>100
	NCI-H23	3.14			>100	>100
	NCI-H322M	9.60			>100	>100
	NCI-H460	3.12			>100	>100
	NCI-H522	1.57			4.39	>100
**Colon cancer**	COLO 205	9.18	4.43	1.33	>100	>100
	HCC-2998	6.68			>100	>100
	HCT-116	2.49			>100	>100

	HCT-15	2.12			>100	>100
	HT29	NT			>100	NT
	KM12	3.08			30.30	>100
	SW-620	3.03			>100	>100
**CNS cancer**	SF-268	4.37	2.85	2.06	>100	>100
	SF-295	2.60			>100	>100
	SF-539	2.14			5.04	80.30
	SNB-19	3.27			72.40	>100
	SNB-75	1.17			5.92	>100
	U251	3.52			>100	>100
**Melanoma**	LOX IMVI	2.93	3.09	1.90	>100	>100
	MALME-3M	2.76			>100	>100
	M14	2.90			>100	>100
	MDA-MB-435	0.65			3.33	79.10
	SK-MEL-2	2.18			6.98	>100
	SK-MEL-28	7.07			>100	>100
	SK-MEL-5	2.42			26.70	>100
	UACC-257	4.37			>100	>100
	UACC-62	2.52			>100	>100
**Ovarian cancer**	IGROV1	2.94	4.05	1.45	>100	>100
	OVCAR-3	2.58			9.93	95.30
	OVCAR-4	6.78			>100	>100
	OVCAR-5	8.18			>100	>100
	OVCAR-8	3.60			>100	>100
	NCI/ADR-RES	1.74			6.54	>100
	SK-OV-3	2.56			>100	>100
**Renal cancer**	786-0	NT	23.91	0.25	NT	NT
	A498	>100			>100	>100
	ACHN	4.08			>100	>100
	CAKI-1	2.04			>100	>100
	RXF 393	2.36			>100	>100
	SN12C	4.13			>100	>100
	TK-10	52.70			>100	>100
	UO-31	2.03			>100	>100
**Prostate cancer**	PC-3	3.38	8.53	0.69	>100	>100
	DU-145	5.15			>100	>100

**Breast cancer**	MCF7	3.07	2.36	2.49	>100	>100
	MDA-MB-231/ATCC	2.87			23.50	>100
	HS 578T	1.81			7.28	>100
	BT-549	2.58			>100	>100
	T-47D	3.44			>100	>100
	MDA-MB-468	0.39			0.00	>100
**Mid** [a]	5.87					

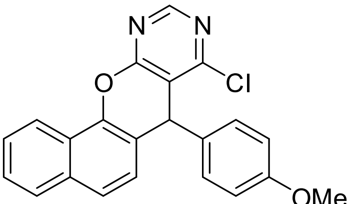

3a

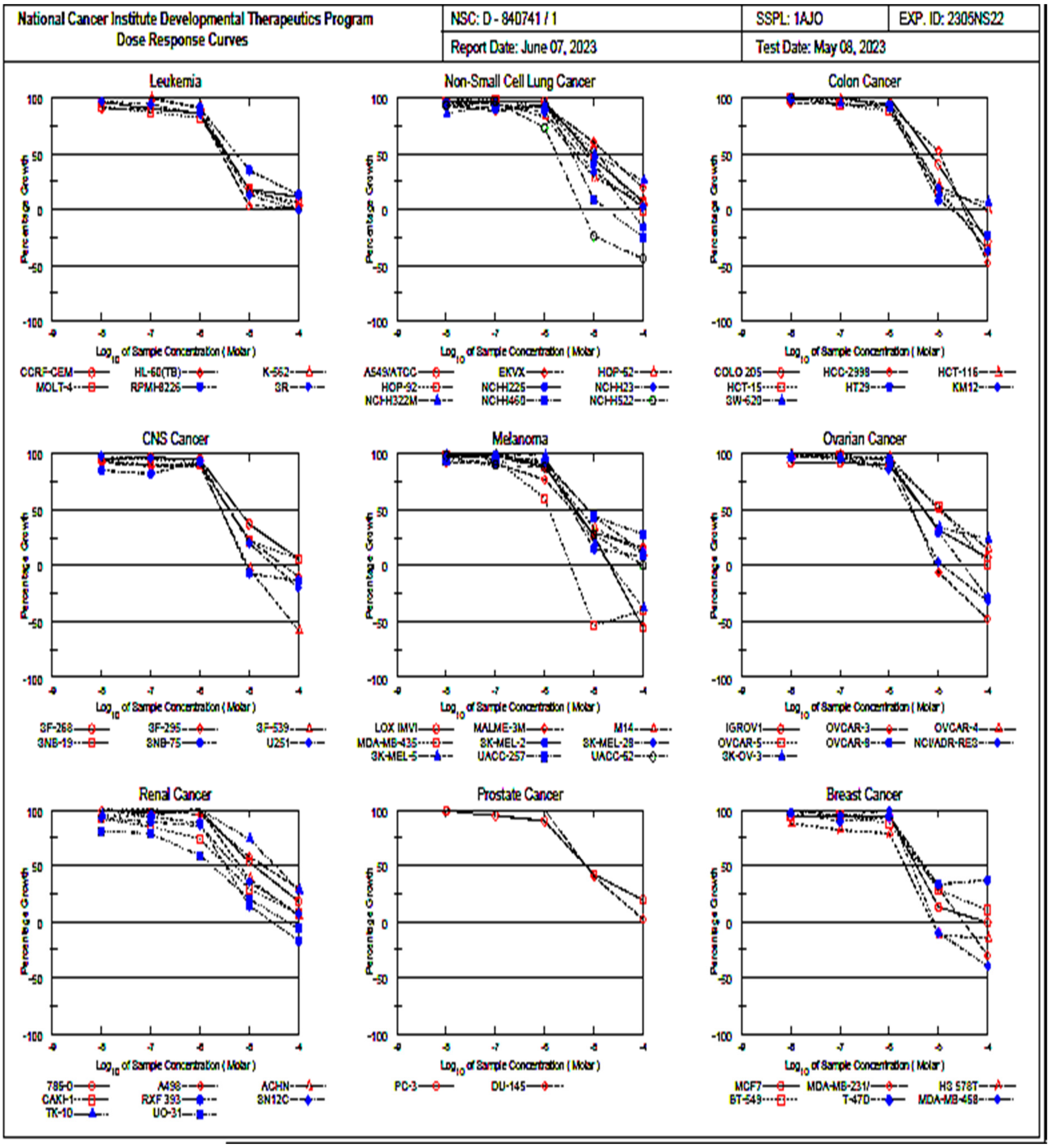

Figure S1: Dose response curves of compound **3a** against 60 cancer cell lines.

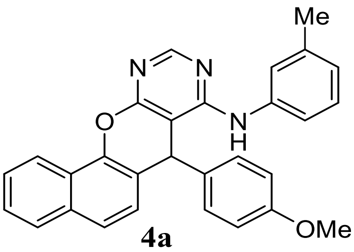

4a

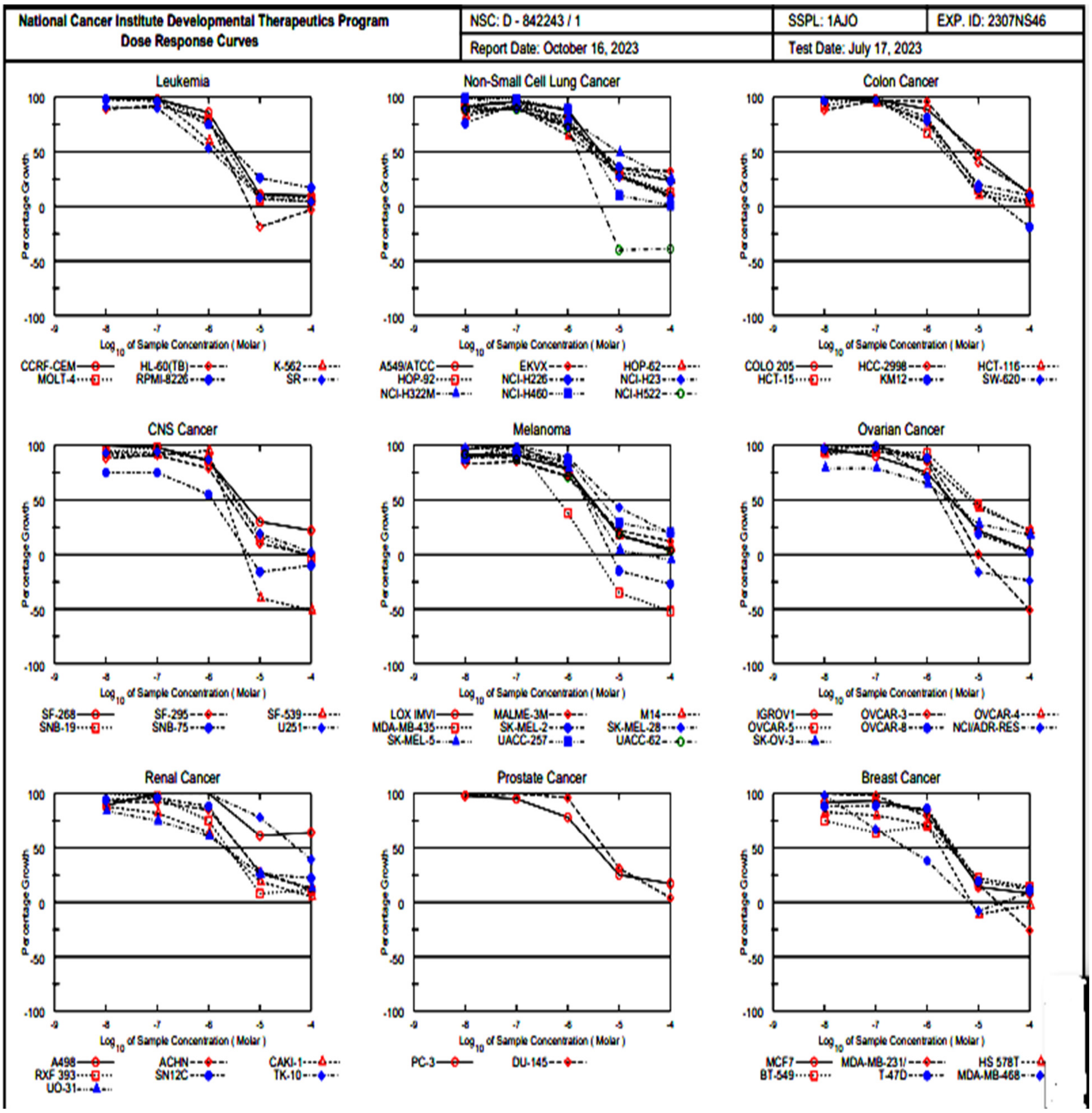

Figure S2: Dose response curves of compound **4a** against 60 cancer cell lines.

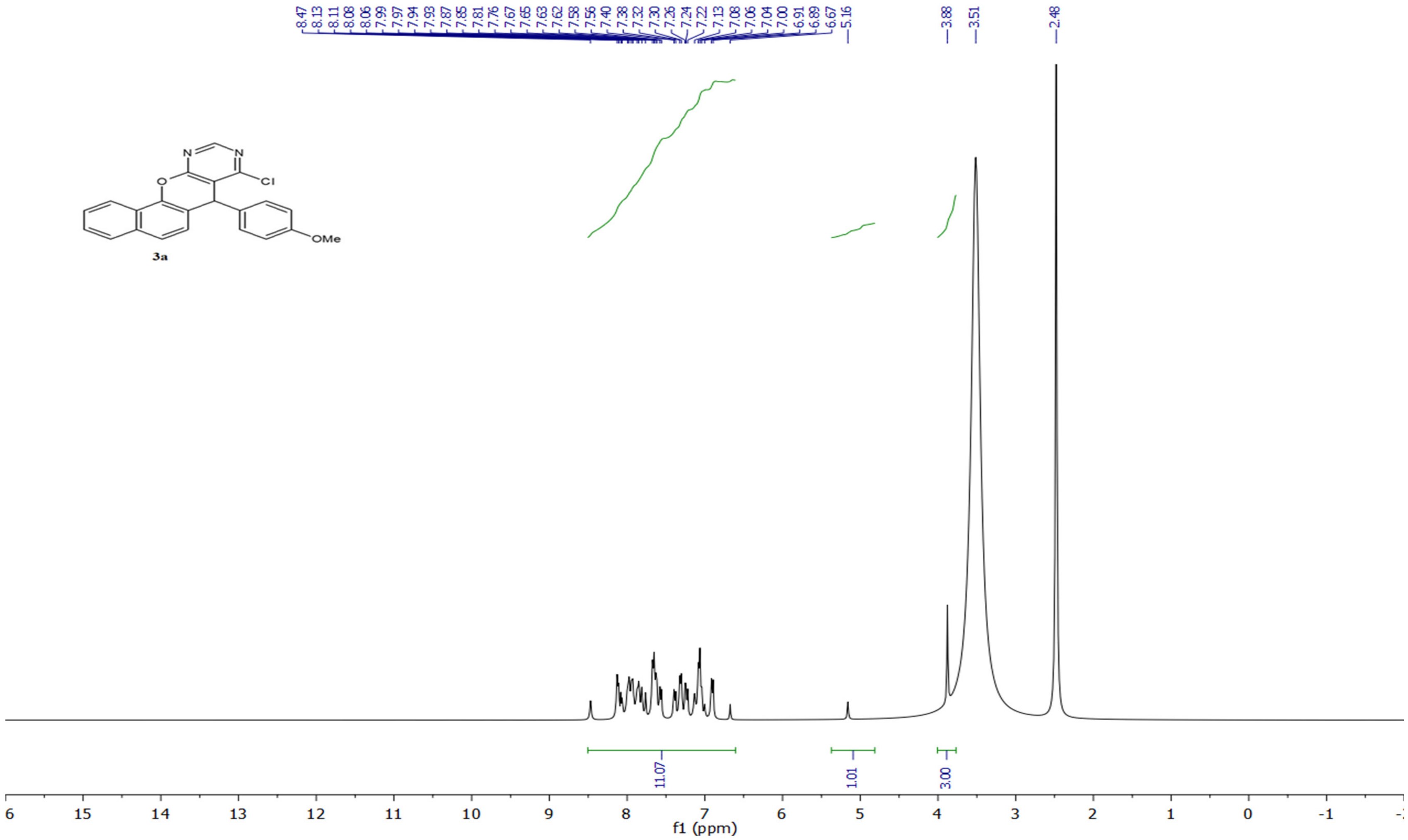

Figure S3: ^{1}H-NMR spectrum of compound **3a** DMSO-d_6

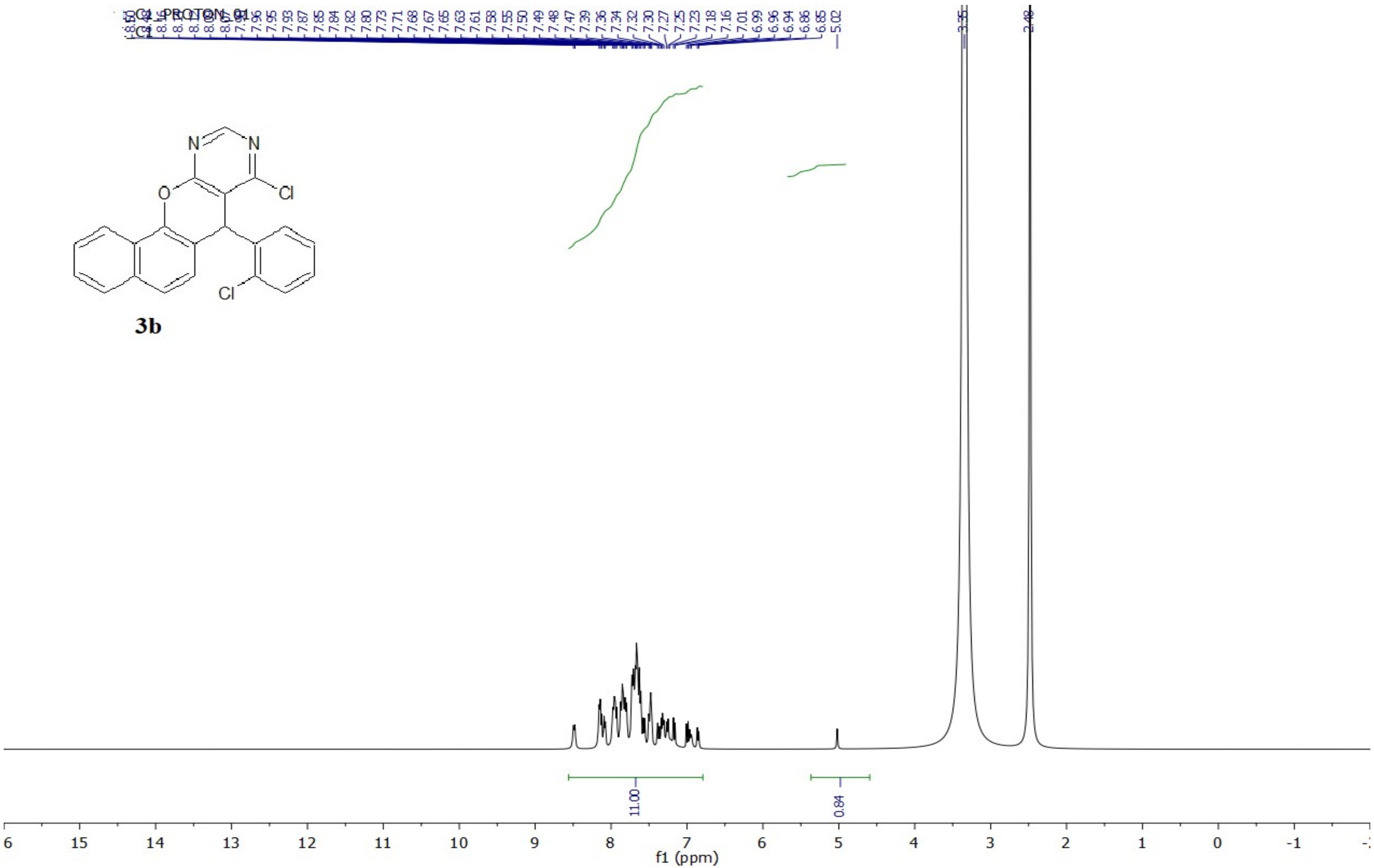

Figure S4: ^{1}H-NMR spectrum of compound **3b** DMSO-d_6.

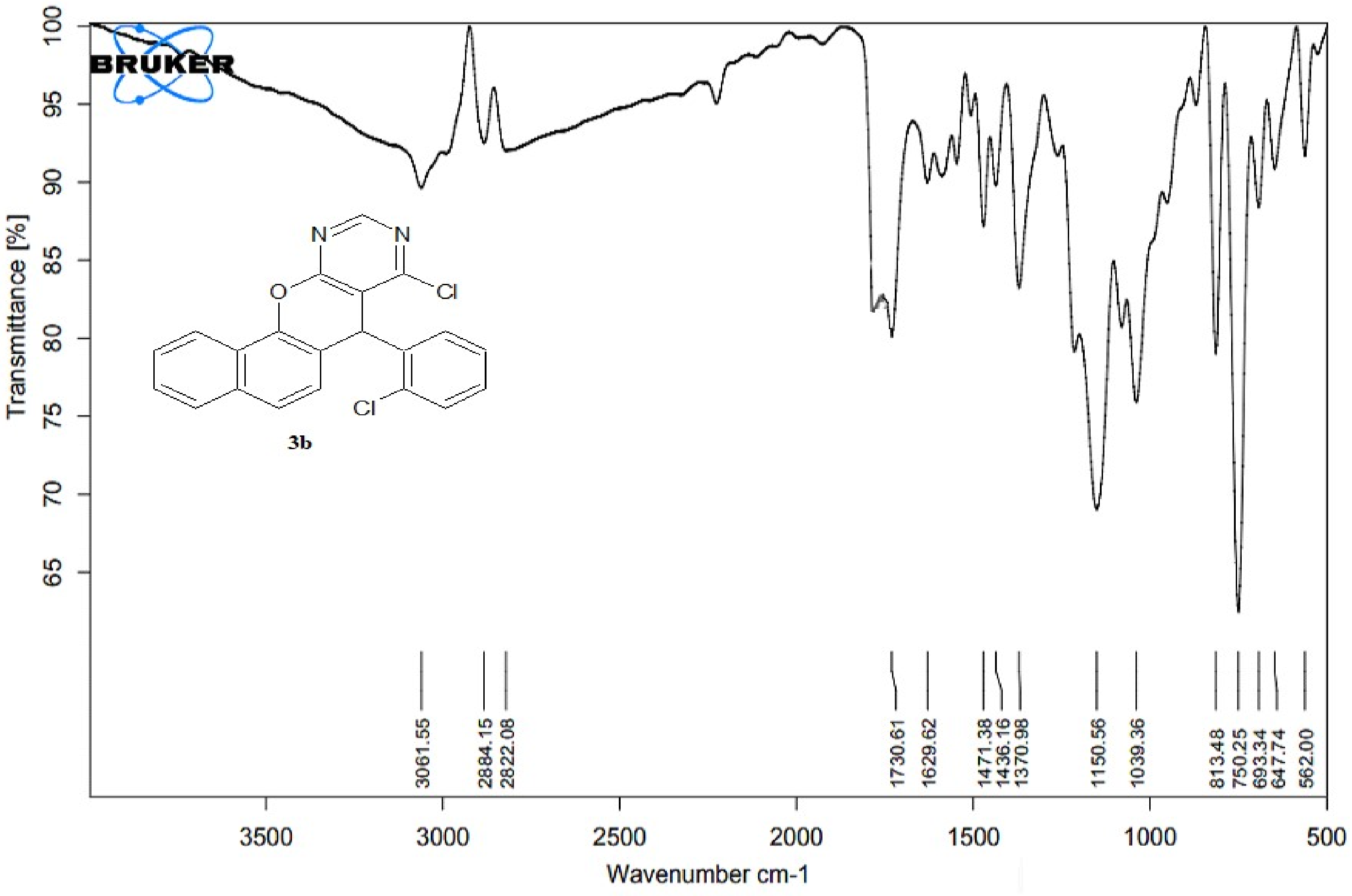

Figure S5: IR spectrum of compound **3b.**

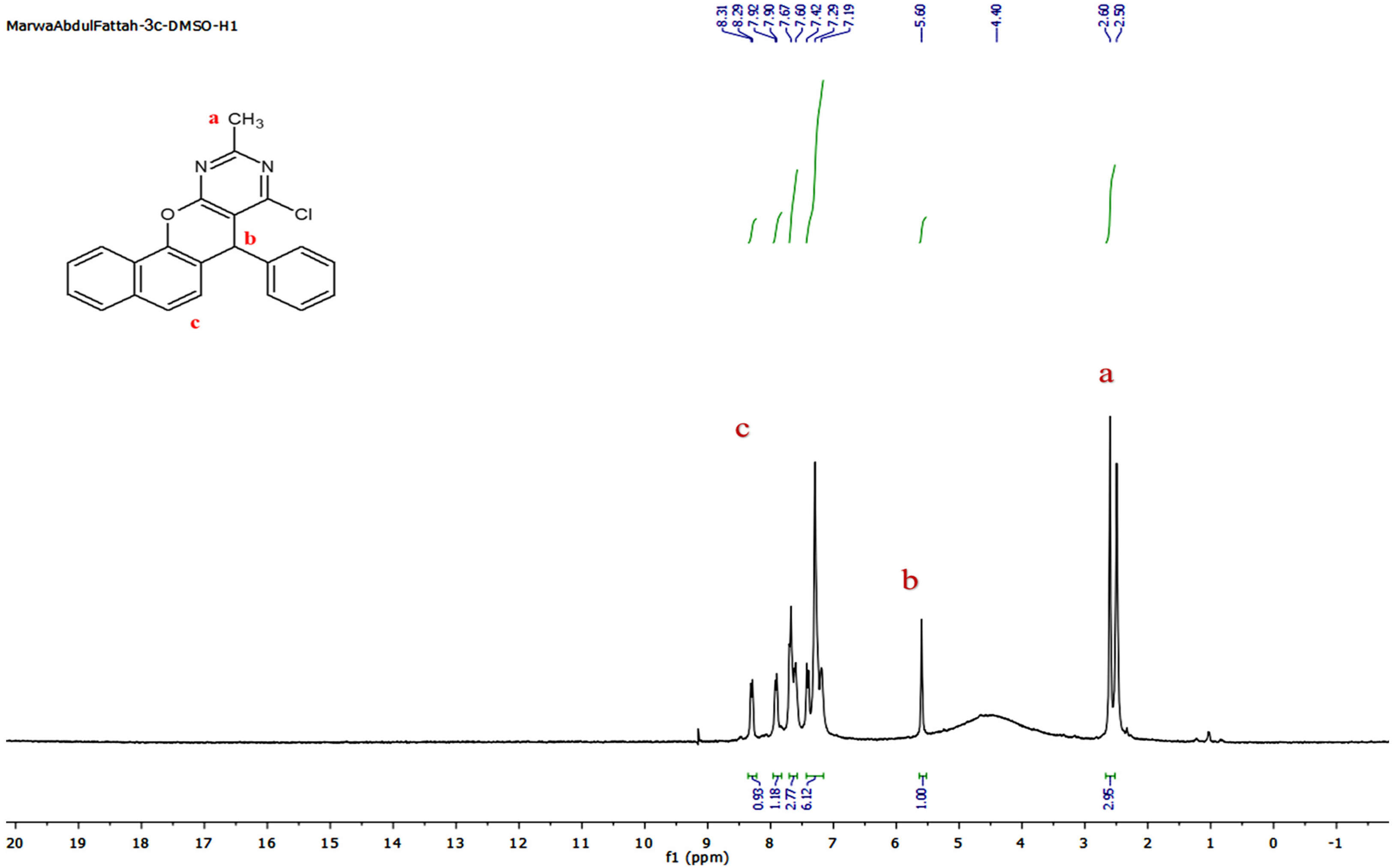

Figure S6: ^{1}H-NMR spectrum of compound **3c** DMSO-*d6.*

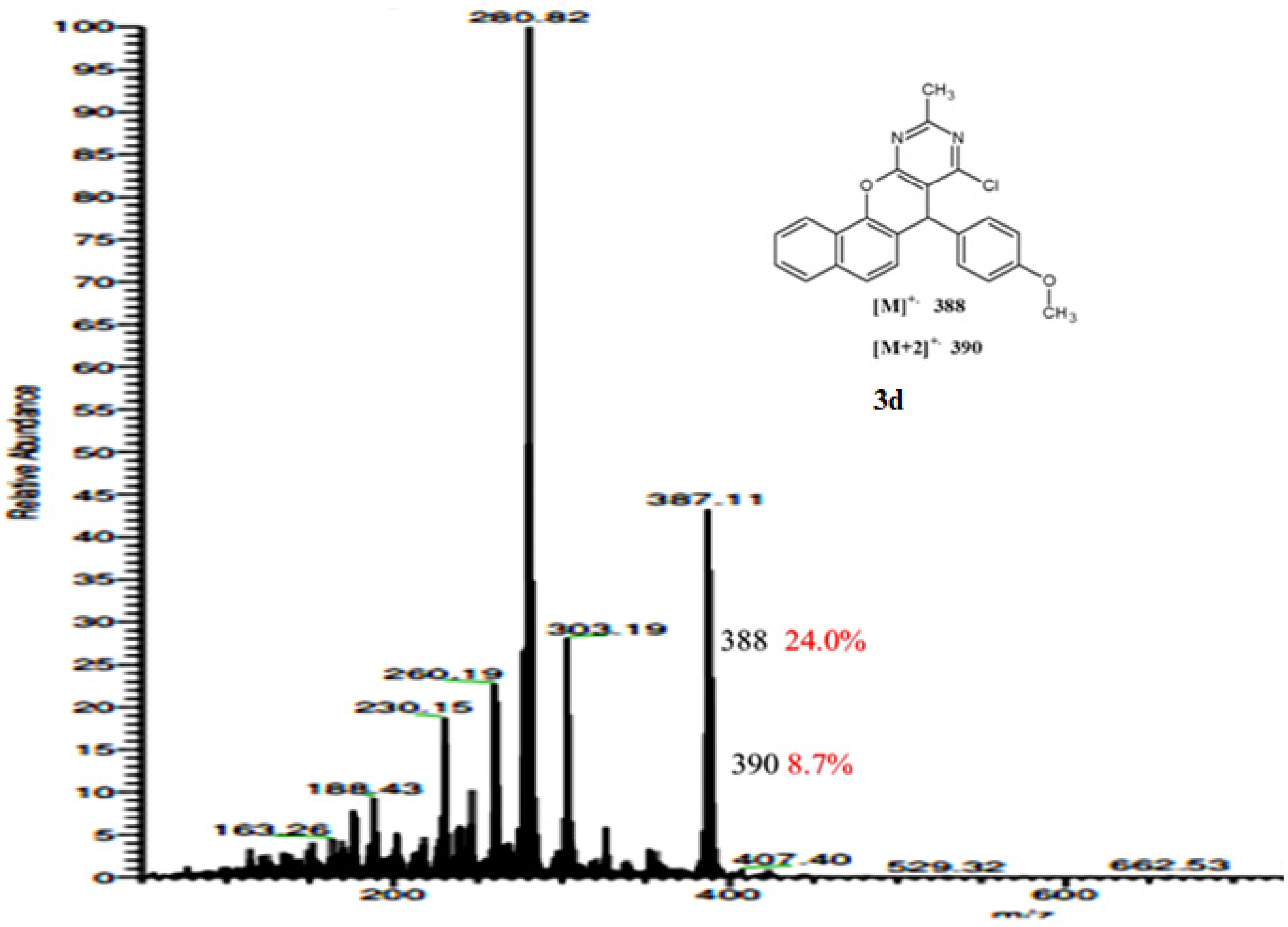

Figure S7: Mass spectrum of compound **3d.**

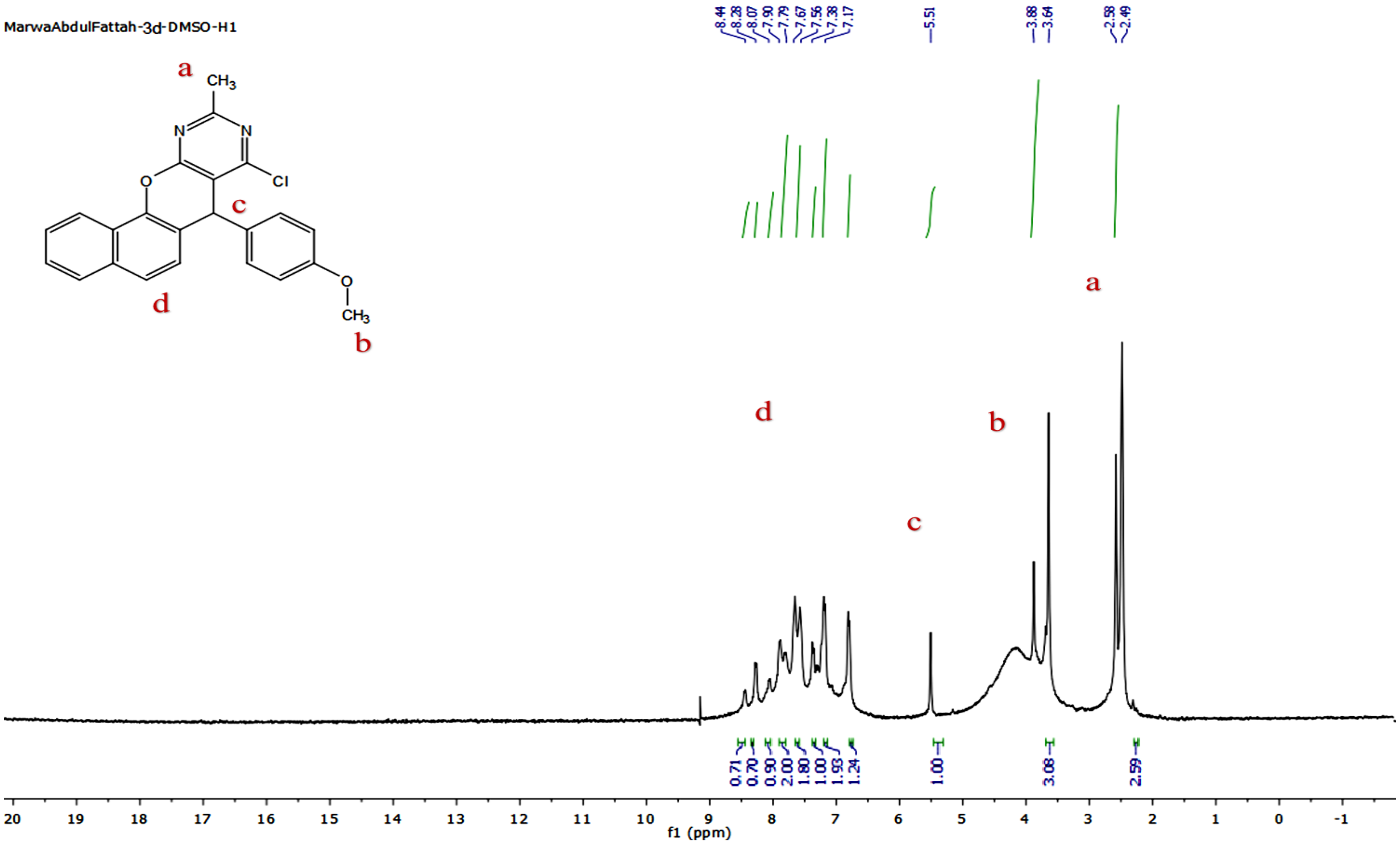

Figure S8: ^{1}H-NMR spectrum of compound **3d** DMSO-*d6.*

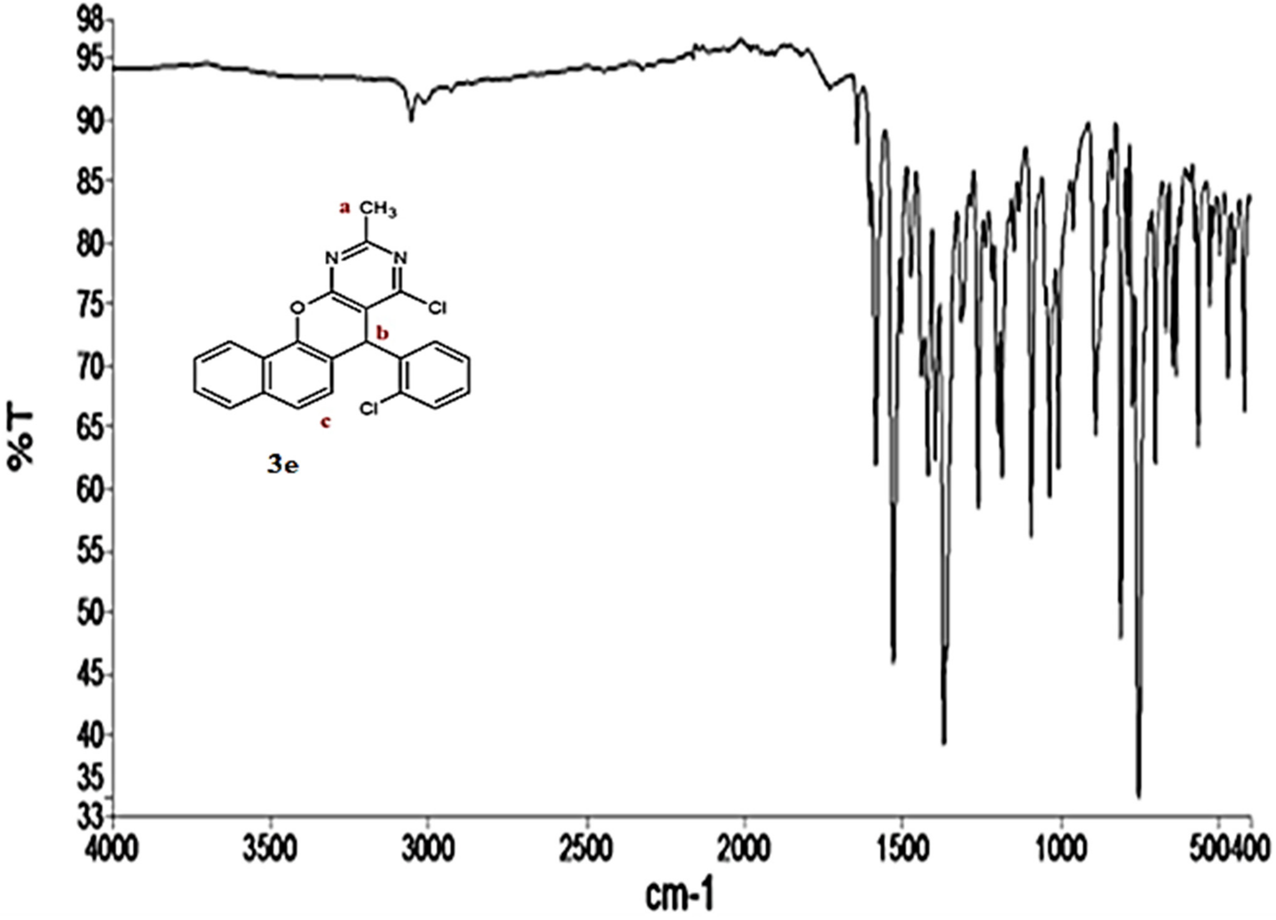

Figure S9: IR spectrum of compound **3e.**

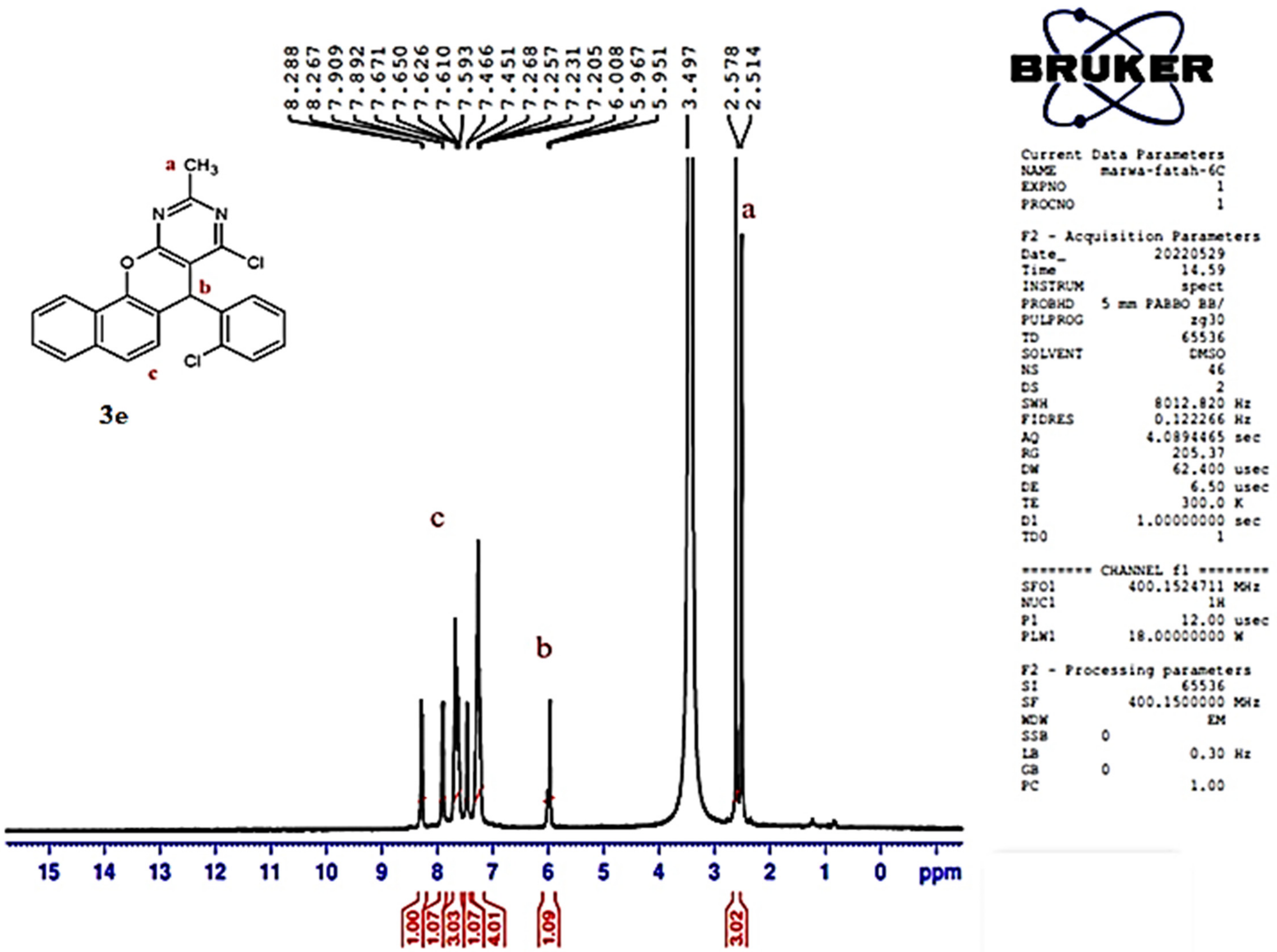

Figure S10: ^{1}H-NMR spectrum of compound **3e** DMSO-*d*6.

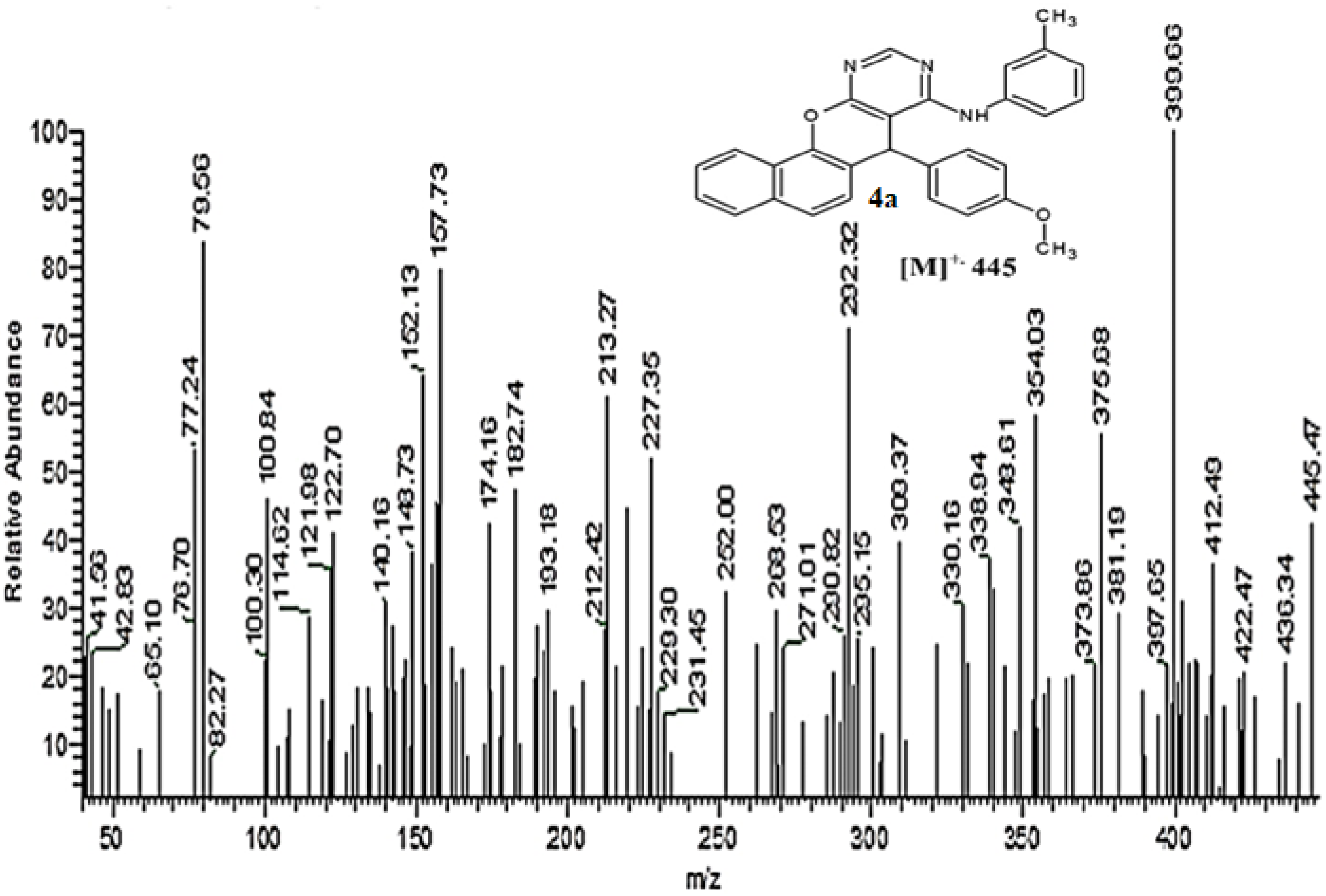

Figure S11: Mass spectrum of compound **4a.**

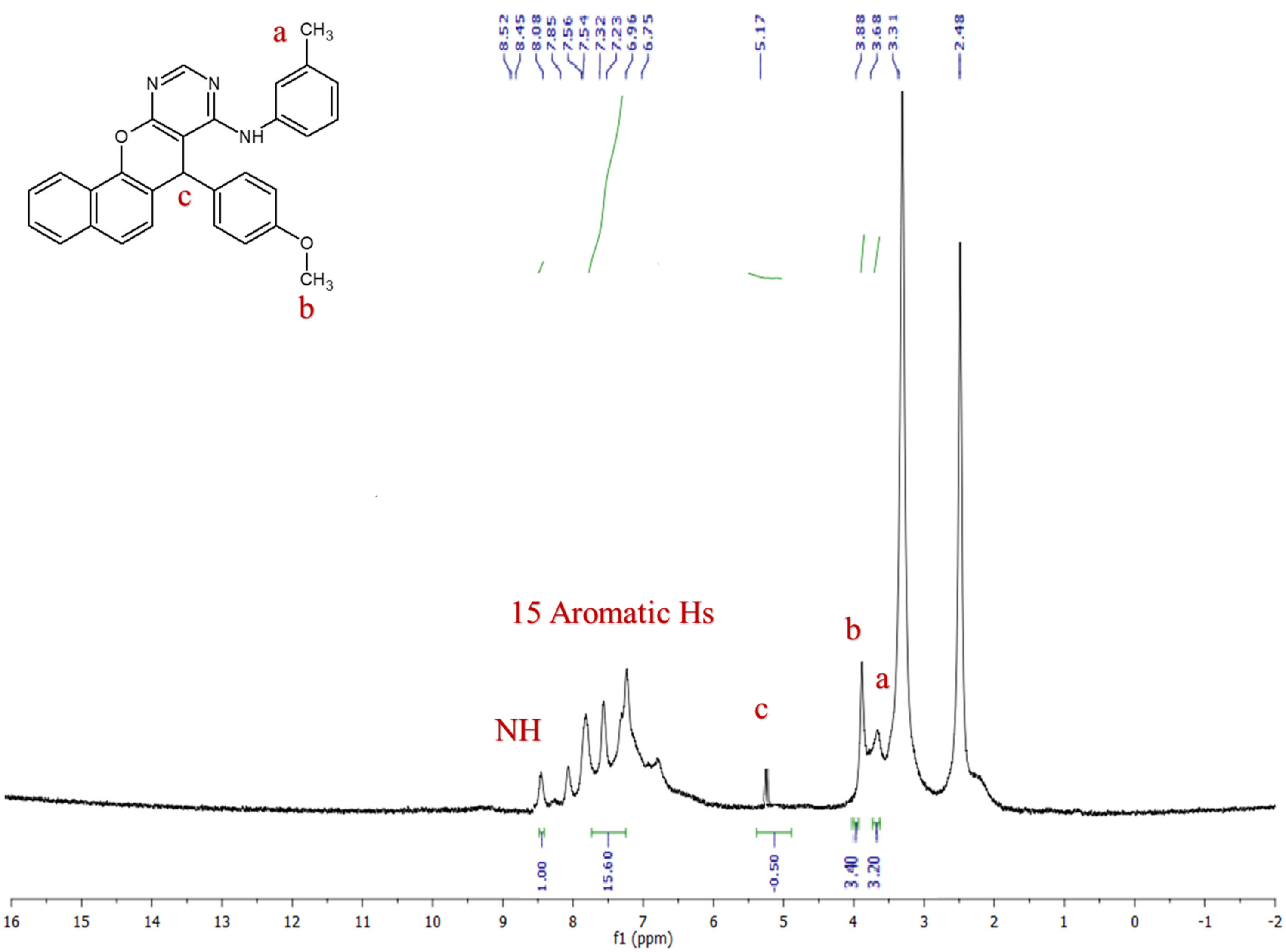

Figure S12: ^{1}H-NMR spectrum of compound **4a** DMSO-*d*6.

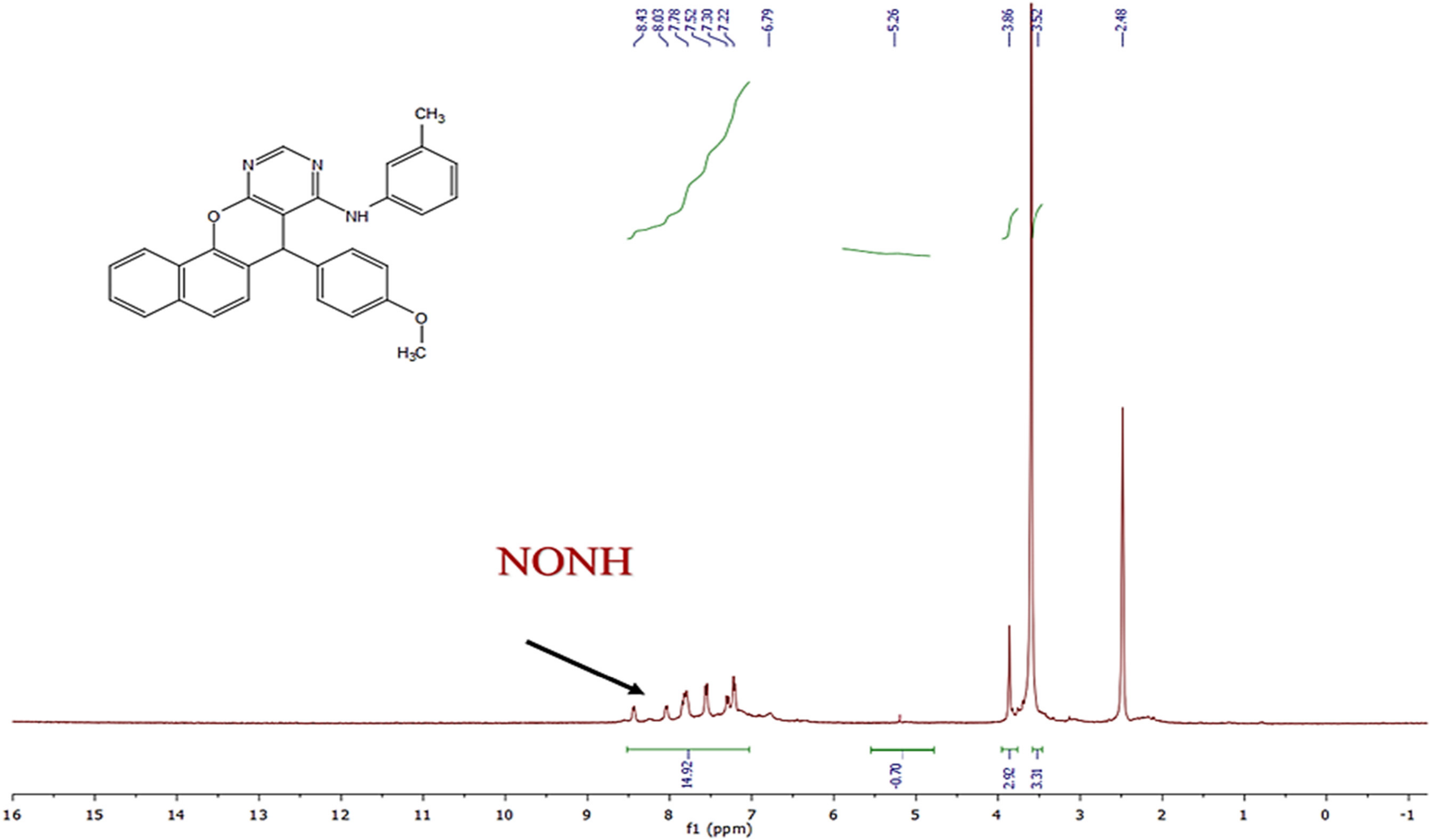

Figure S13: ^{1}H-NMR spectrum of compound **4a** D_2O.

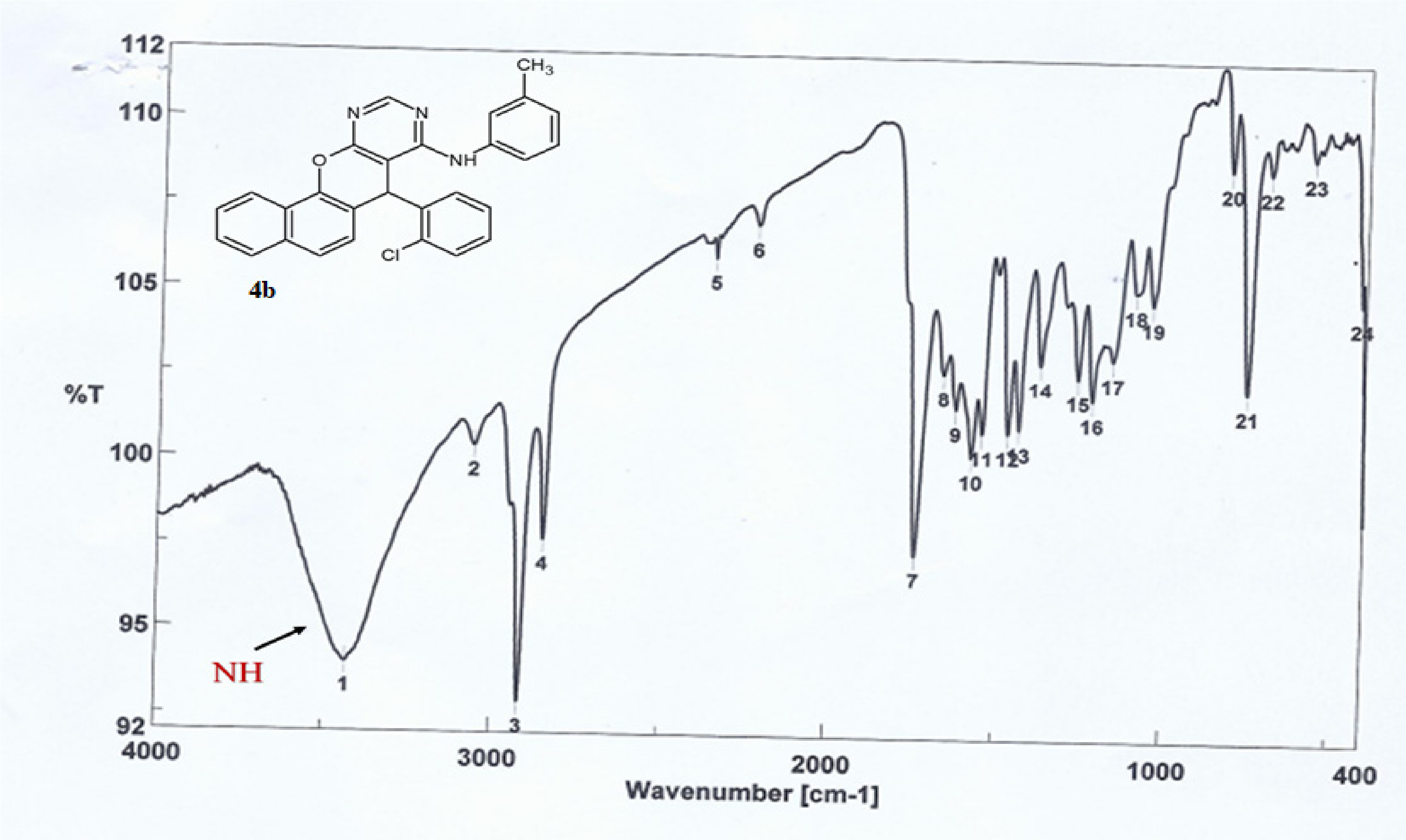

Figure S14: IR spectrum of compound **4b.**

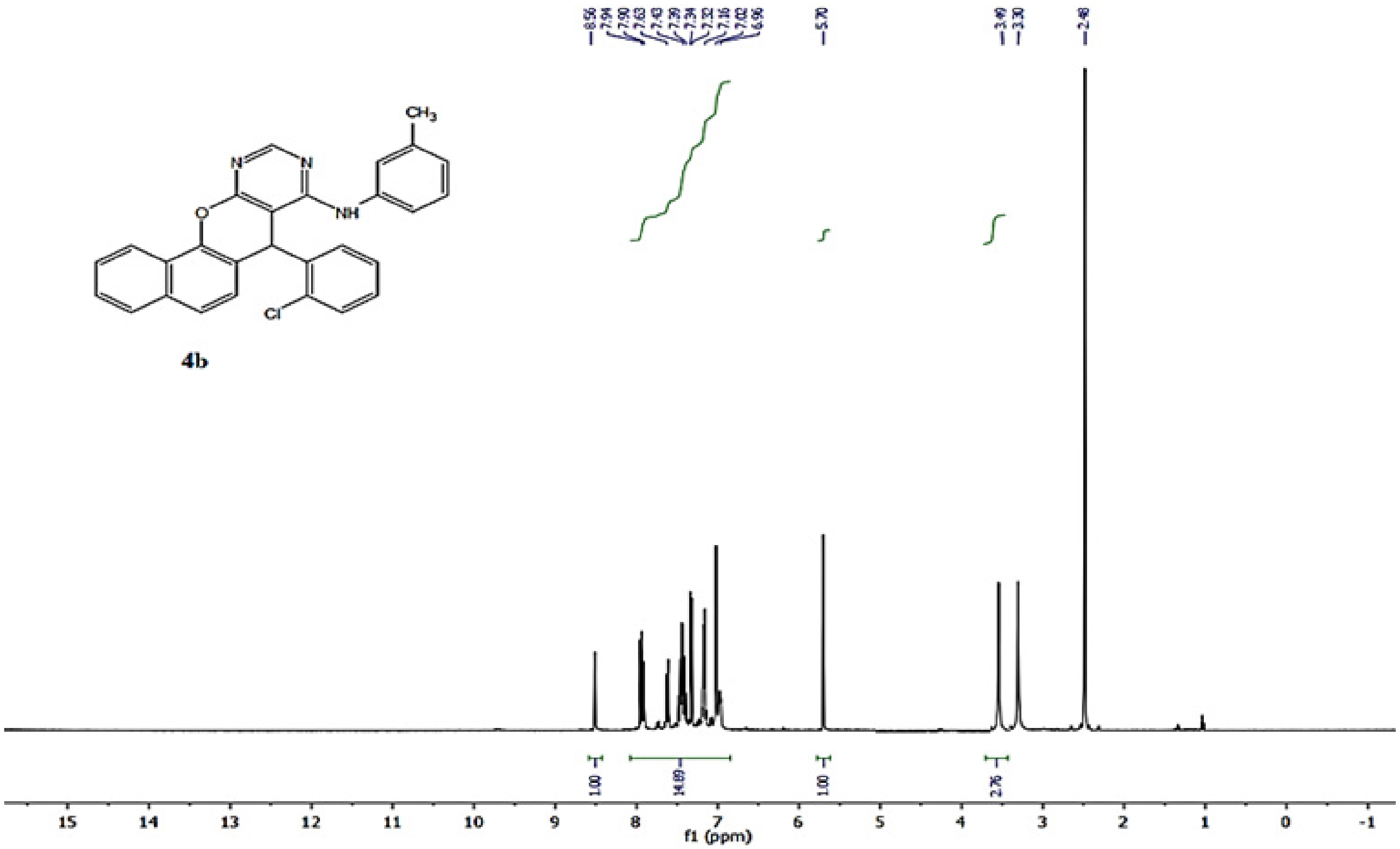

Figure S15: ^{1}H-NMR spectrum of compound **4b** DMSO-d_6.

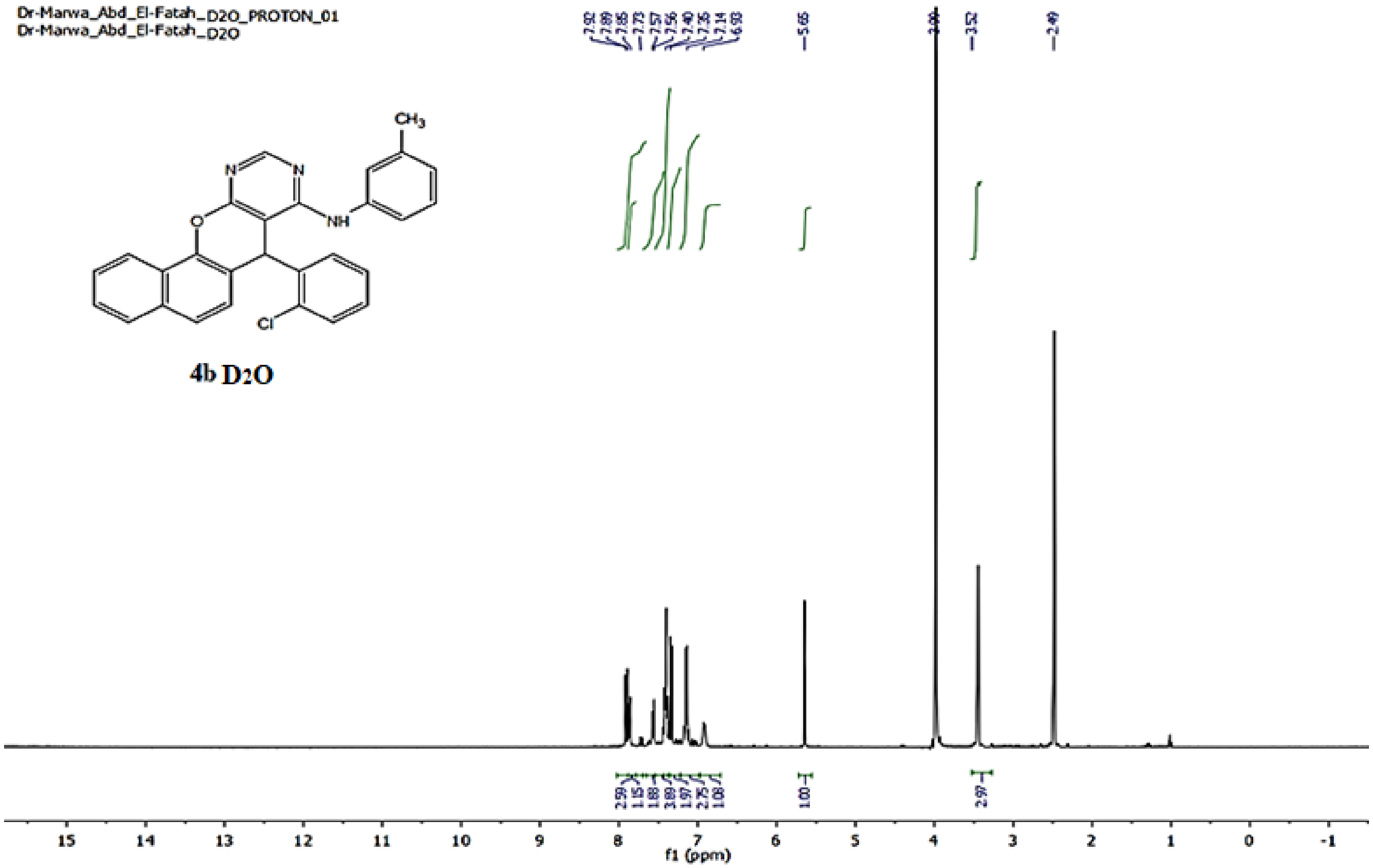

Figure S16: ^{1}H-NMR spectrum of compound **4b** D_2O.

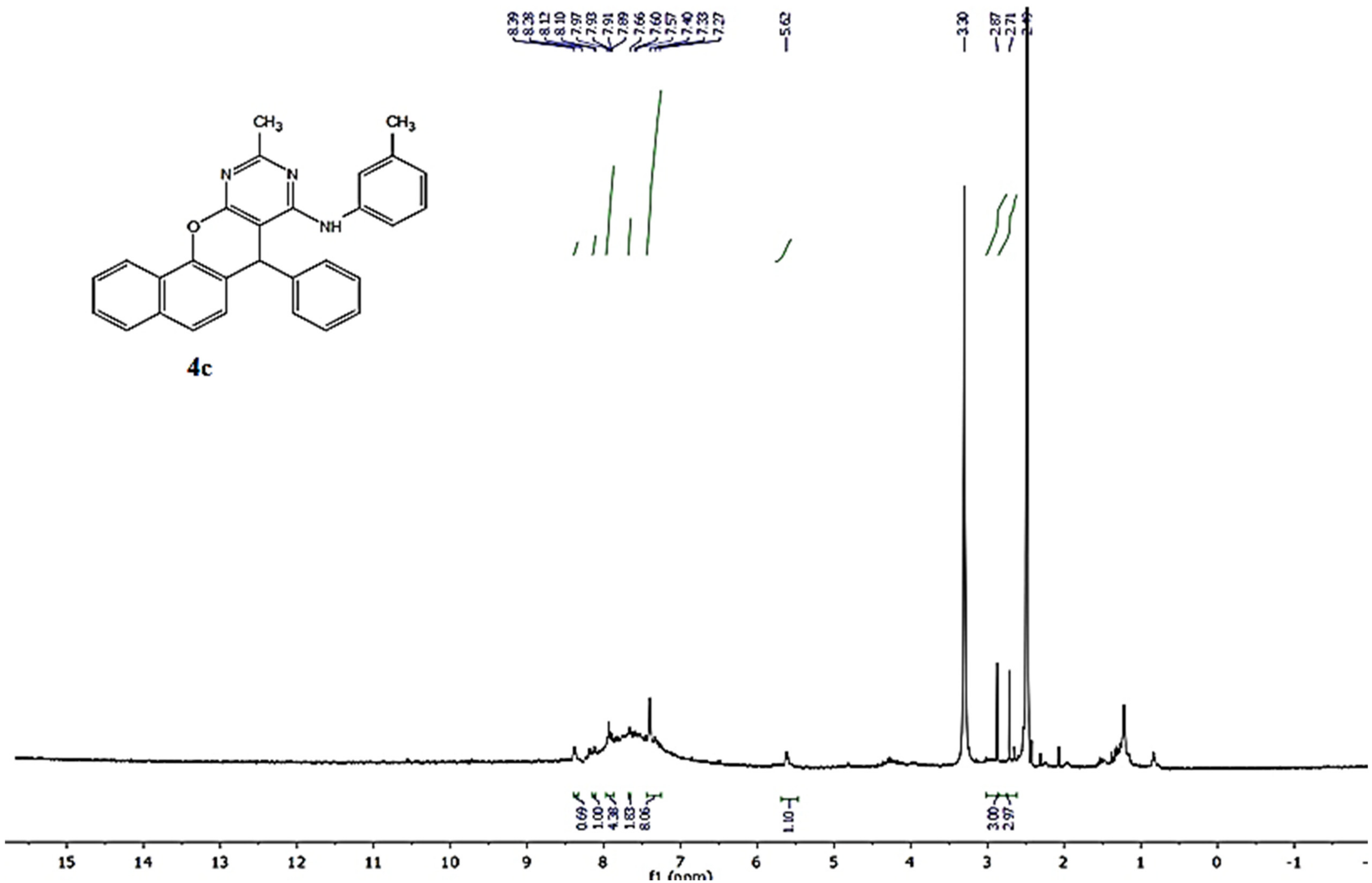

Figure S17: ^{1}H-NMR spectrum of compound **4c** DMSO-d_6.

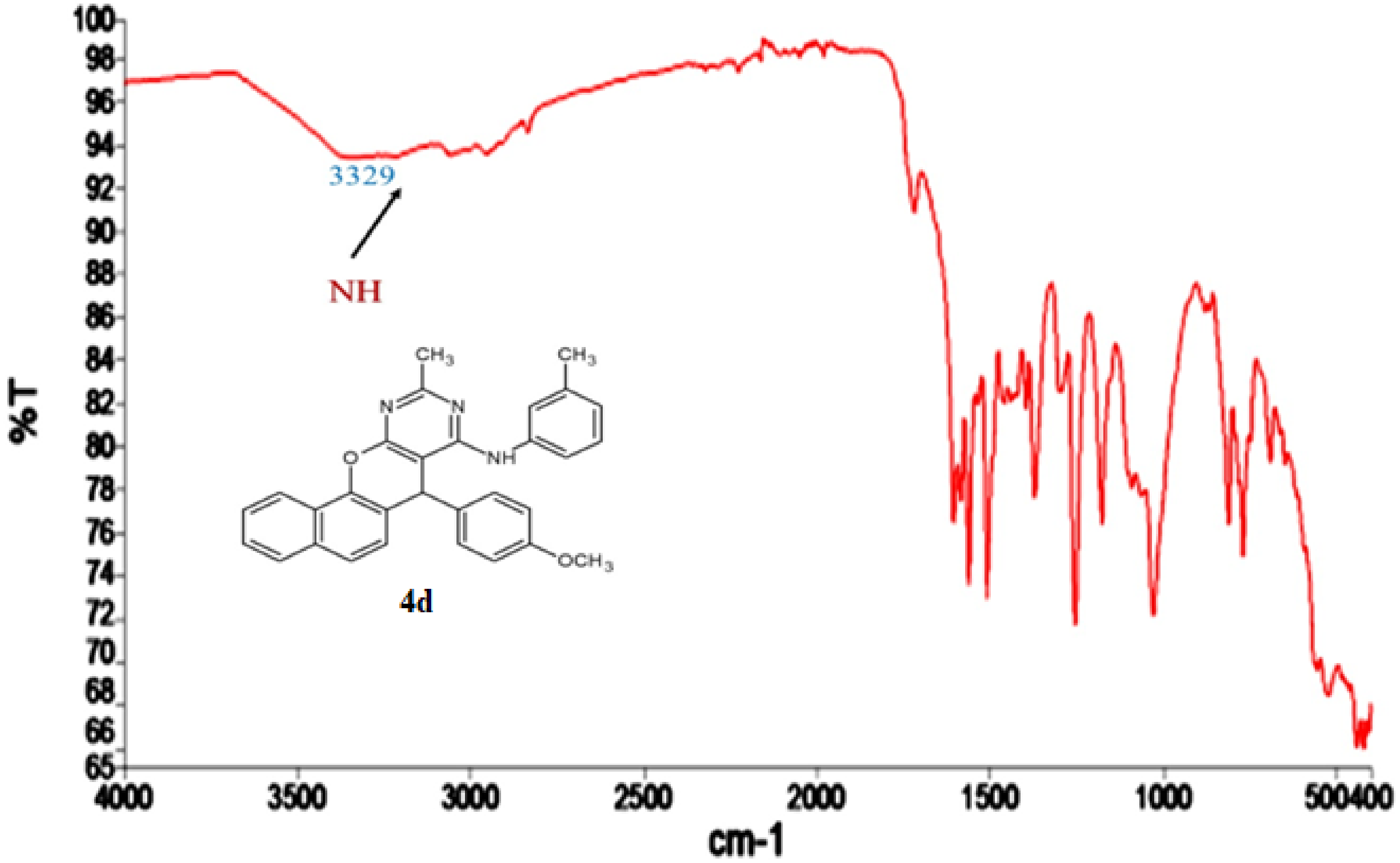

Figure S18: IR spectrum of compound **4d.**

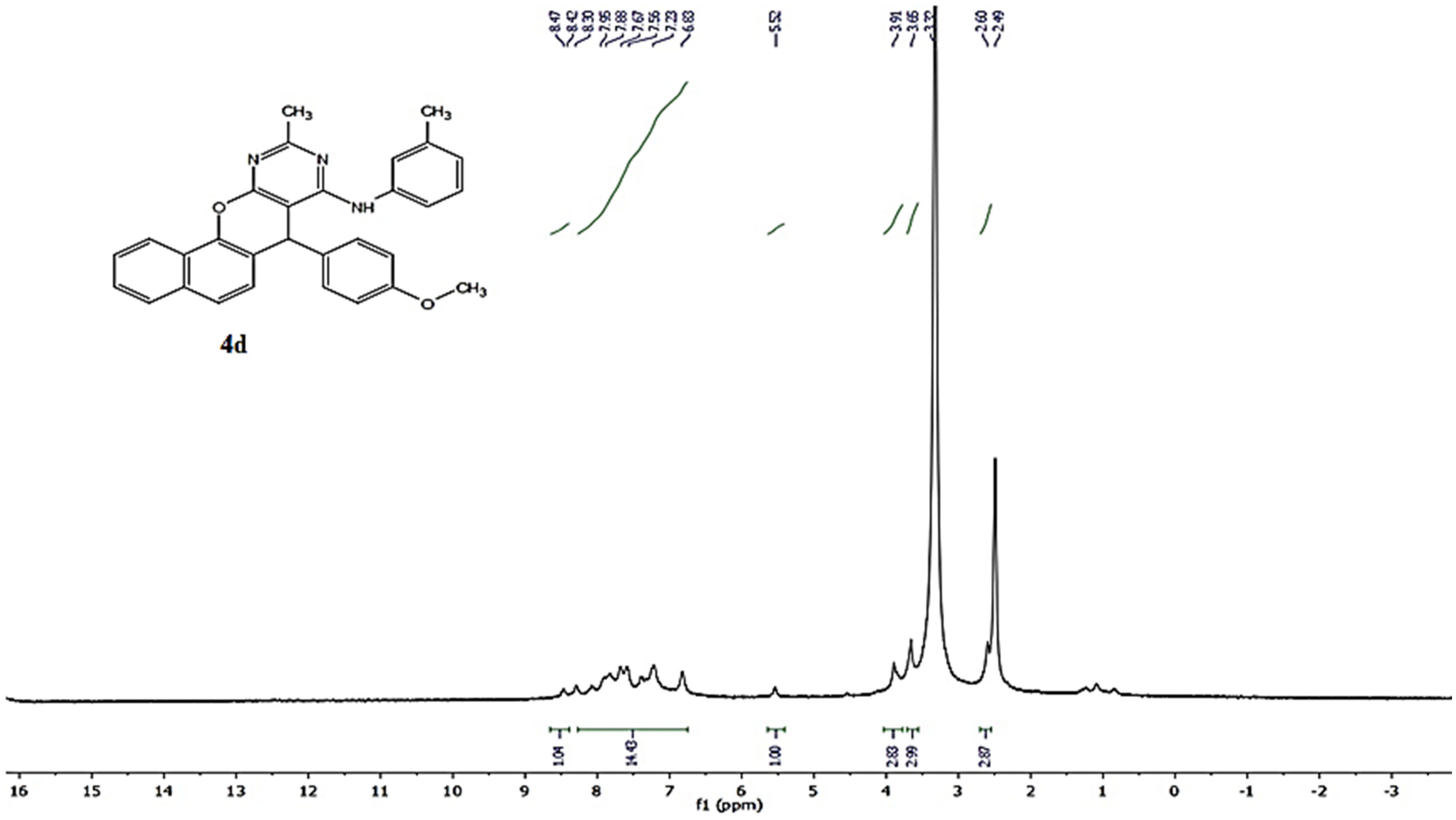

Figure S19: ^{1}H-NMR spectrum of compound **4d** DMSO-d_6.

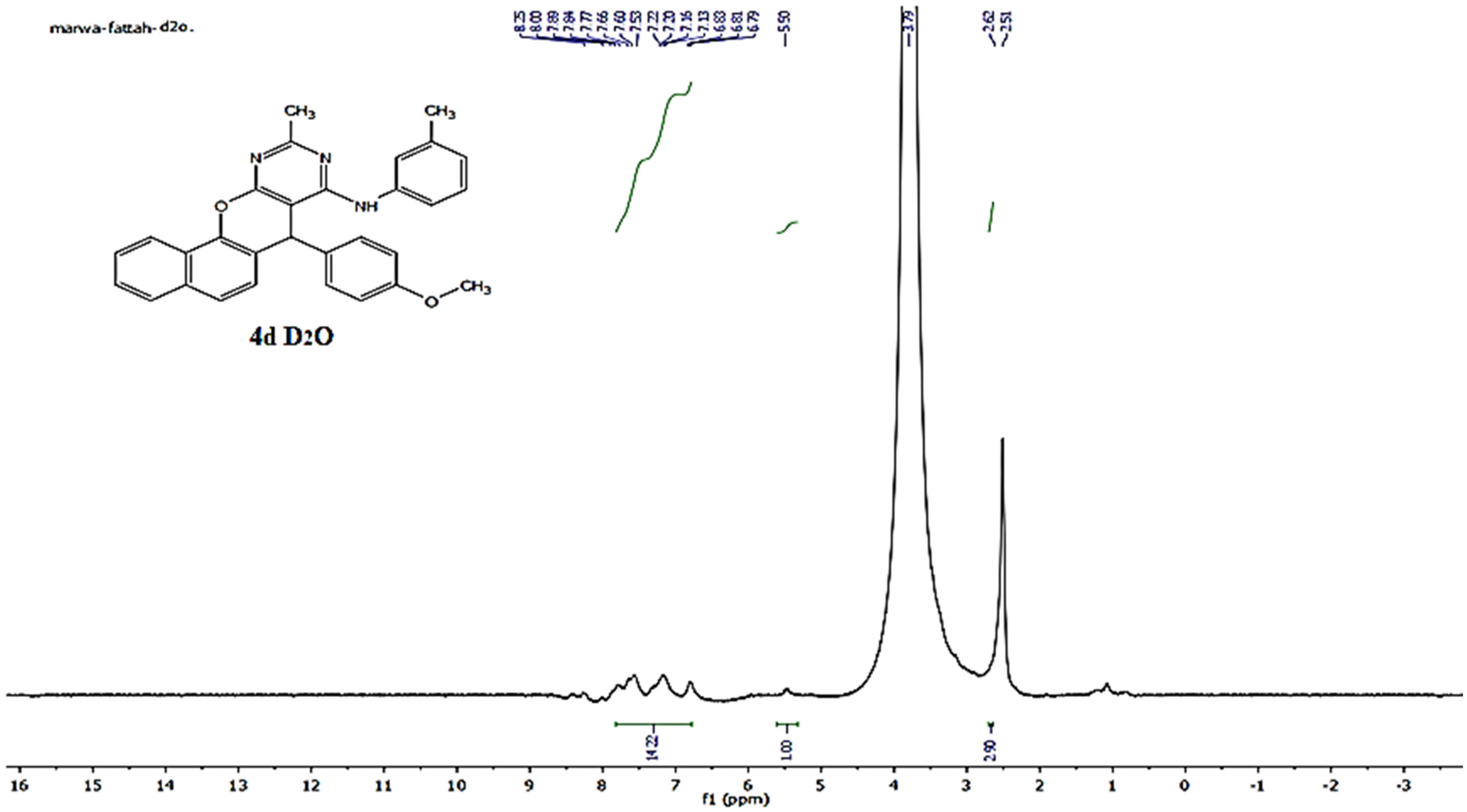

Figure S20: ^{1}H-NMR spectrum of compound **4d** D_2O.

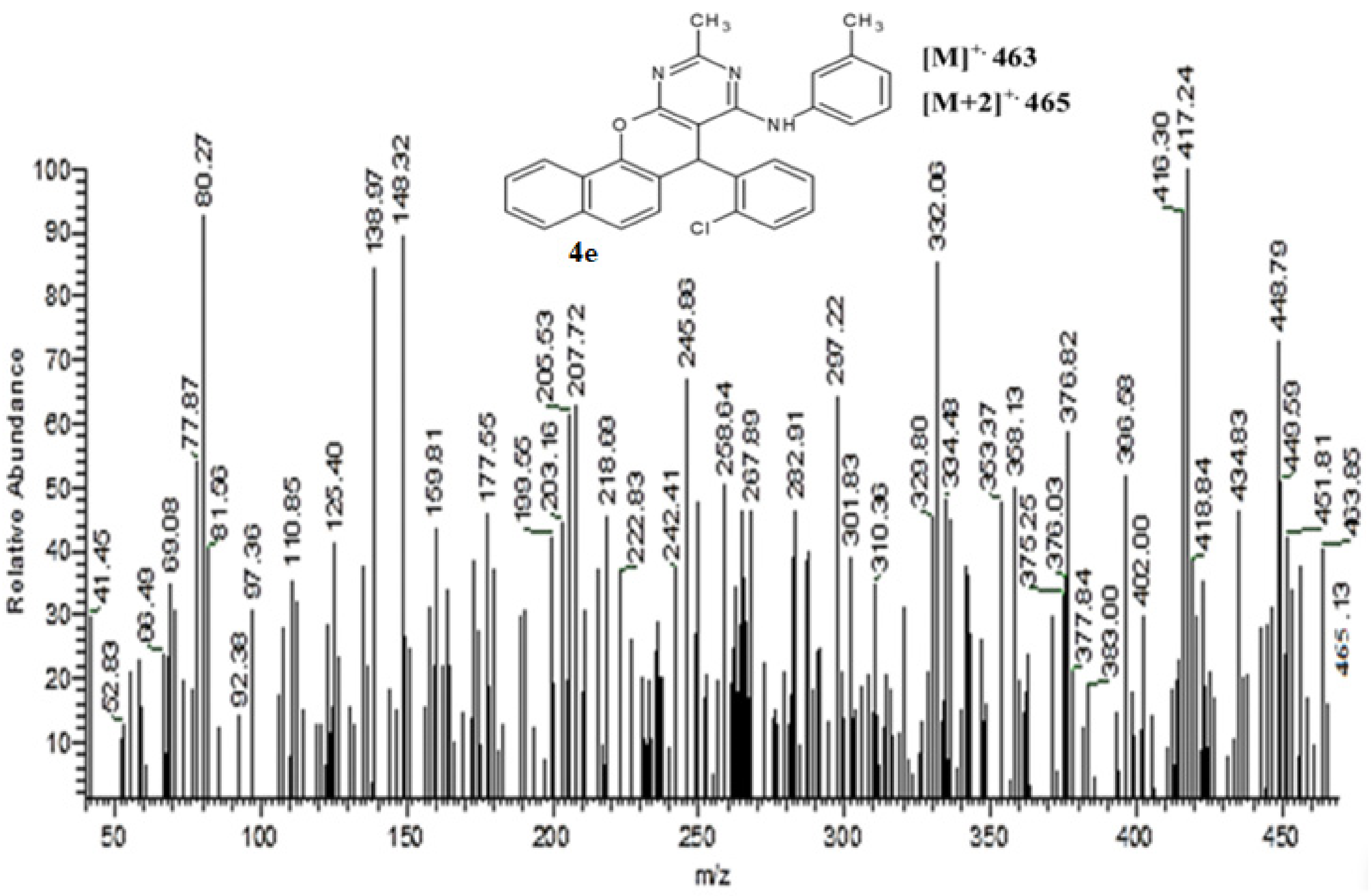

Figure S21: Mass spectrum of compound **4e.**

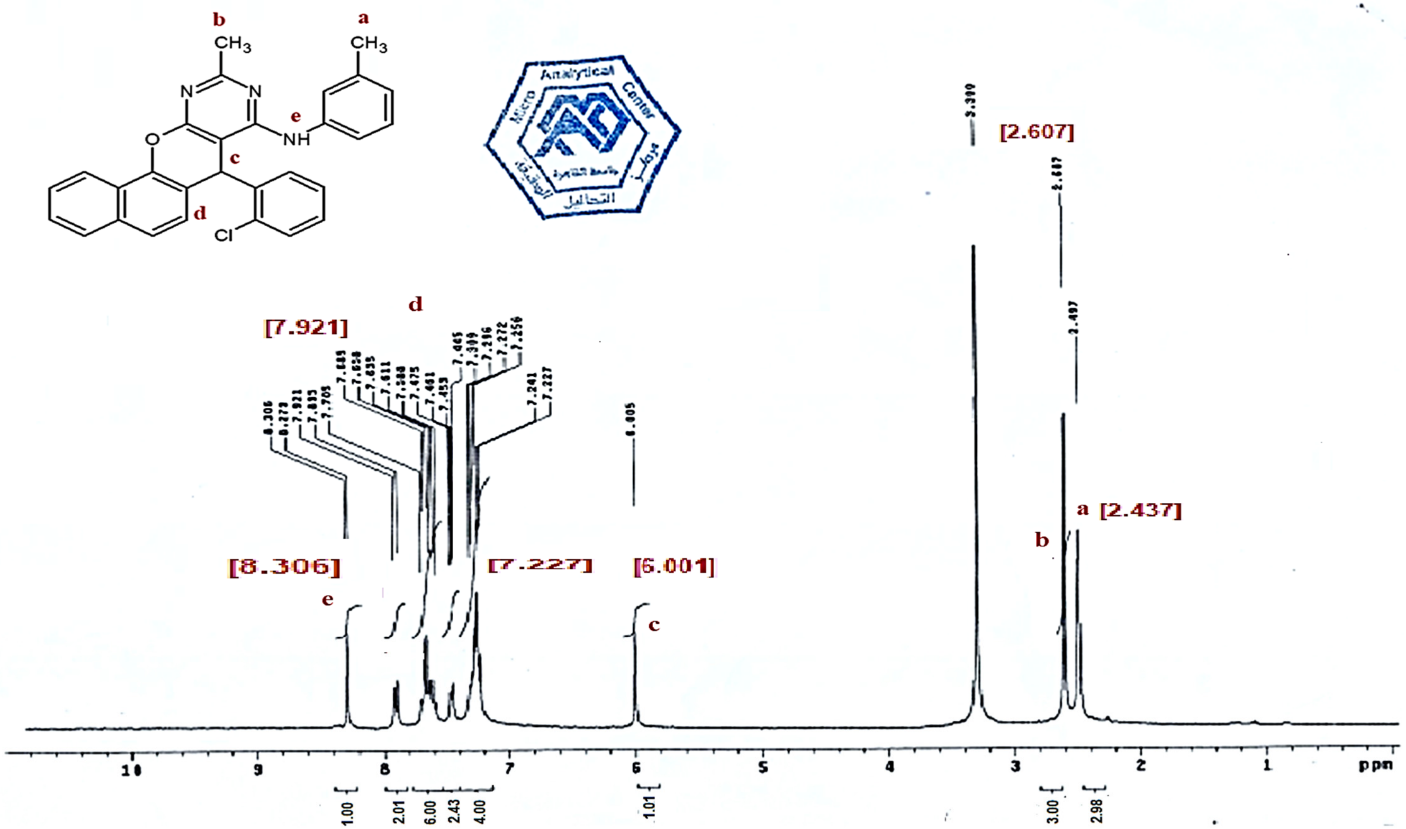

Figure S22: ^{1}H-NMR spectrum of compound **4e** DMSO-*d*$_{6}$.

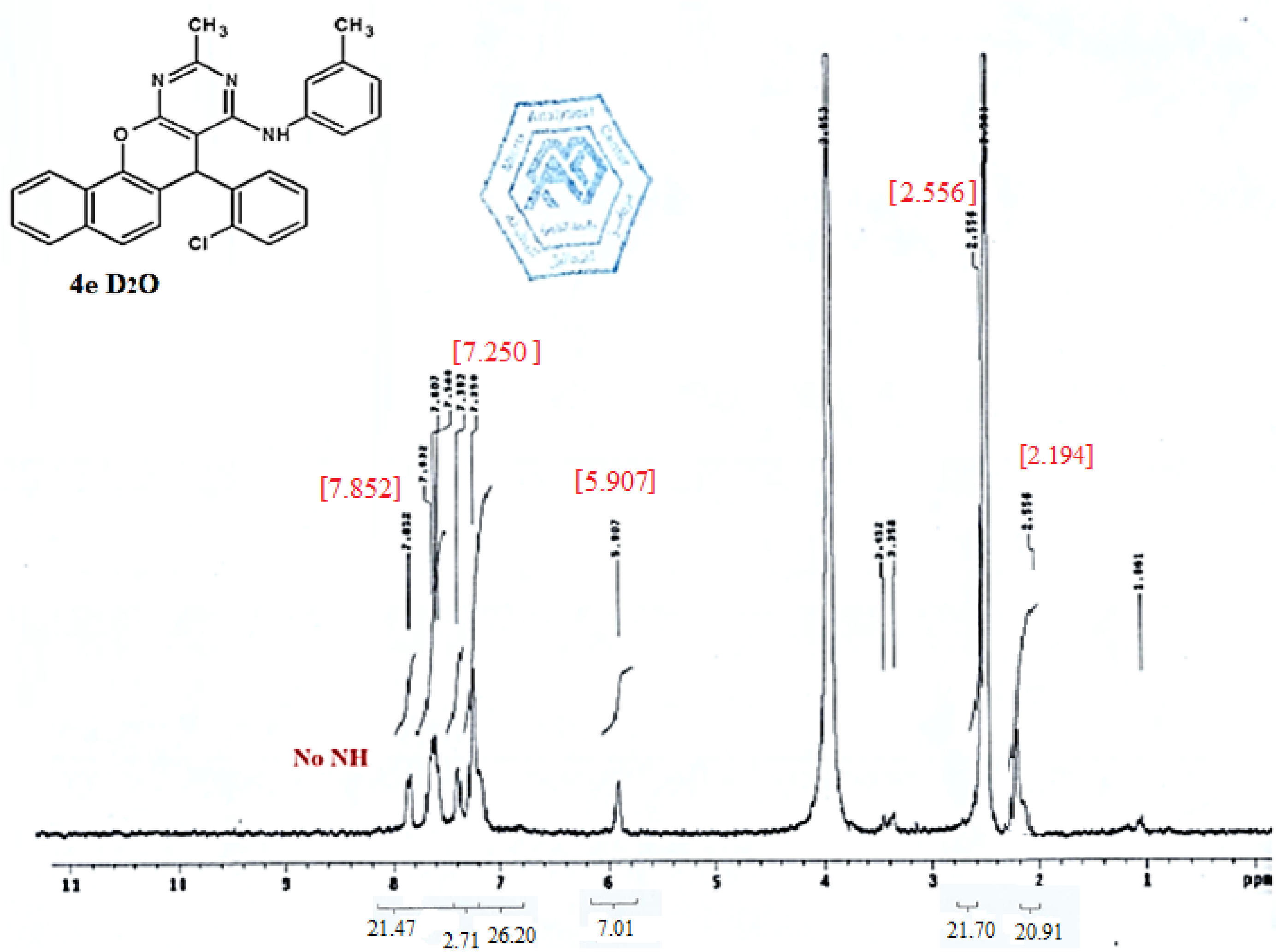

Figure S23: ^{1}H-NMR spectrum of compound **4e** D_2O.
